# Tumor-targeted Exosomal Delivery of Celastrol for Enhanced Therapeutic Efficacy in NSCLC

**DOI:** 10.7150/thno.125096

**Published:** 2026-03-03

**Authors:** Disha Nagesh Moholkar, Raghuram Kandimalla, Mohd Saeed, Yaseera Arif, Neha Tyagi, Richa Singhal, Al-Hassan Kyakulaga, Amir Saeed, Margaret Wallen, Ramesh Gupta, Farrukh Aqil

**Affiliations:** 1 Brown Cancer Center, University of Louisville, Louisville, KY 40202, USA.; 2 Department of Medicine, University of Louisville, Louisville, KY 40202, USA.; 3 Department of Pharmacology and Toxicology, University of Louisville, Louisville, KY 40202, USA.; 4 Department of Biology College of Sciences, University of Hail, Hail, Saudi Arabia.; 5 Center for Cardiometabolic Science, University of Louisville, Louisville, KY 40202, USA.; 6 Department of Clinical Laboratory Sciences, College of Applied Medical Sciences, University of Hail, Hail, Saudi Arabia.; 7 3P Biotechnologies Inc., Louisville, KY 40202, USA.

**Keywords:** Exosomes, Oral targeted delivery, Epithelial-to-mesenchymal transition (EMT), Chemoresistance, Celastrol, Lung cancer.

## Abstract

**Rationale:**

Non-small cell lung cancer (NSCLC) continues to impose a significant global mortality burden, due to limited therapies, drug resistance, and treatment-related toxicity. Exosomes offer promise for the targeted delivery of therapeutic agents.

**Methods:**

Exosomes were isolated from bovine colostrum and characterized for size, polydispersity index, and surface charge. Celastrol (CEL) was loaded onto exosomes (ExoCEL), and Folic Acid (FA)-functionalized exosomes (FA-ExoCEL) and validated using fluorescence quenching and protease sensitivity assay. Anticancer activity was assessed in NSCLC cell lines using colony formation, cell migration and uptake assays. Transcriptomic (RNA-seq) and protein analysis were performed to analyze gene expression changes. Biodistribution, oral uptake and potential toxicity were evaluated in wild-type mice, while oral antitumor efficacy was tested in orthotopic lung tumor models comparing CEL, ExoCEL and FA-ExoCEL. Synergistic activity with paclitaxel was assessed in chemoresistant cells.

**Results:**

Exosomes were isolated, characterized and efficiently loaded with CEL. ExoCEL demonstrated superior antiproliferative effects in NSCLC cell lines and enhanced potency in drug-resistant A549TR cells compared to free CEL. ExoCEL significantly inhibited colony formation and cell migration in a dose-dependent manner. RNA Seq and protein analyses showed that CEL and ExoCEL reversed TGF-β-induced EMT, restored epithelial markers, suppressed mesenchymal, oncogenic and extracellular matrix related markers. In orthotopic lung tumor models, FA-ExoCEL achieved approximately 80-90% tumor inhibition, outperforming both free CEL and ExoCEL. Oral delivery of FA-ExoCEL resulted in efficient gastrointestinal uptake, selective tumor targeting, recovery of exosomal markers in circulation and no observed systemic toxicity. CEL exhibited strong synergy with paclitaxel, with exosomal delivery further enhancing paclitaxel efficacy in resistant cells.

**Conclusions:**

FA-ExoCEL represents a safe, scalable, and effective oral therapeutic strategy for NSCLC. By combining exosome-mediated delivery with folate-targeted tumor accumulation, this platform enhances CEL bioavailability, and improves antitumor efficacy, supporting its translational potential for lung cancer therapy.

## Introduction

Lung cancer is the second most prevalent cancer and accounts for the highest number of cancer-related deaths worldwide. In the United States alone, over 225,000 new cases and approximately 142,000 deaths from lung cancer are reported annually, with NSCLC accounting for approx. 85% of all cases 1. Unfortunately, approximately 85% of NSCLC patients ultimately die within five years of diagnosis. Even after surgical resection, disease recurrence occurs in 30%-50% of patients 2. Currently, nearly 18 million individuals in the U.S. are cancer survivors, yet only about 3.46% (~654,620) are lung cancer survivors, underscoring the persistent challenges in improving outcomes for this malignancy 3.

Despite advances in targeted therapies, platinum-based chemotherapies, and immune checkpoint inhibitors, survival outcome for patients with NSCLC remains limited. Treatment of early-stage disease typically involves surgical resection and/or radiation in combination with systemic chemotherapy. However, therapeutic options for patients with metastatic disease are limited, and many are ultimately managed with palliative intent to improve quality of life. Relapse, drug resistance, and the debilitating adverse effects of chemo- and radiotherapy highlight the urgent need for safer, more effective treatment modalities.

One promising approach involves the oral delivery of plant-derived therapeutics using nanocarrier systems such as exosomes. Compared to conventional bolus-dose intravenous drug administration, oral delivery offers several advantages, including ease of administration, reduced systemic toxicity, and improved patient compliance and quality of life 4, 5. Throughout human history, plants have played a vital role in traditional and modern medicine. A wide array of plant-derived compounds serves as the basis for evidence-based pharmaceutical drugs. In particular, triterpenoids have shown promise in the treatment of diverse diseases, including cancer. Their broad pharmacological activity, combined with favorable safety profiles, has spurred growing interest in their development as therapeutic agents.

**Figure 1:**
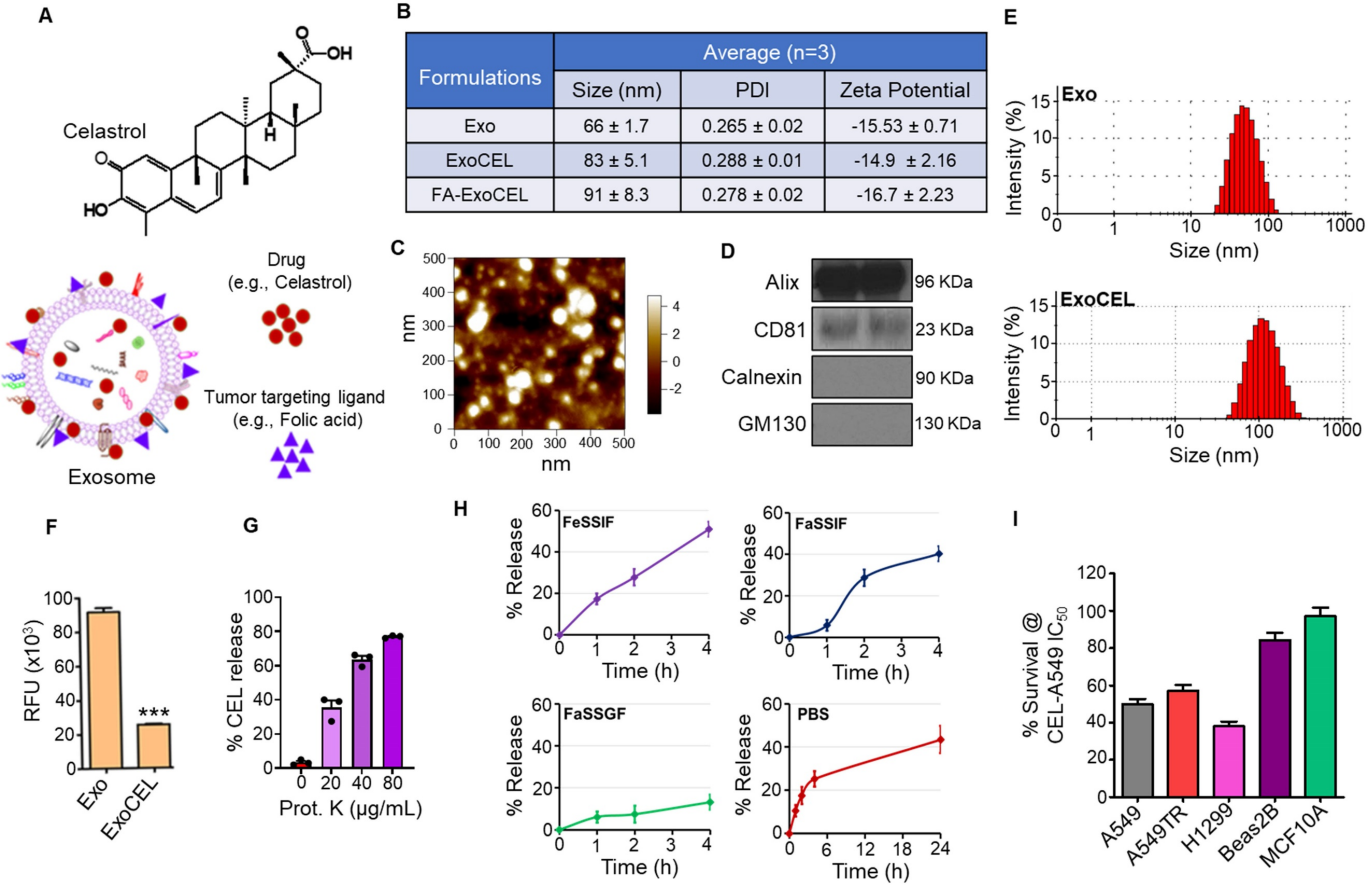
** Characterization of CEL-loaded exosomes (ExoCEL). (A)** Schematic representation of CEL structure and its loading onto bovine colostrum-derived exosomes, with or without folic acid (FA) as a tumor-targeting ligand. **(B)** Physicochemical properties of exosomes (Exo), ExoCEL, and FA-ExoCEL including particle size, polydispersity index (PDI), and zeta potential from three independent batches. **(C)** Atomic force microscopy (AFM) image showing morphology and nanoscale size distribution of exosomes. **(D)** Western blot analysis confirming the presence of exosomal markers (CD81 and Alix) and absence of cellular contaminants (calnexin and GM130). **(E)** Size distribution profiles of unloaded exosomes (Exo) and CEL-loaded exosomes (ExoCEL) analyzed by dynamic light scattering (DLS). **(F)** Fluorescence quenching assay showing a significant reduction in intrinsic protein fluorescence following CEL and FA loading, indicating strong hydrophobic interactions. **(G)** Proteinase K-mediated digestion showing concentration-dependent loss of CEL from ExoCEL, indicating that a substantial fraction of the drug is associated with exosomal surface proteins. **(H)** *In vitro* release profiles of CEL from FA-ExoCEL in biorelevant media, including FeSSIF, FaSSIF, FaSSGF, and PBS, demonstrating time-dependent CEL release behavior in different media. **(I)** Cell viability of NSCLC cells (A549, H1299, A549TR) and normal cells (Beas2B, MCF10A) treated with CEL at its IC₅₀ concentration for A549. CEL selectively inhibited cancer cell survival with minimal effects on normal cells.

Celastrol (CEL) is quinone methide triterpenoid (Figure 1A), a bioactive phytochemical, derived from roots of medicinal plant *Tripterygium wilfordii*. Multiple preclinical studies have shown that triterpenoids, particularly CEL, affect signaling pathways that have oncogenic functions. CEL regulates signal transducer and activator of transcription 3 (STAT3) expression by inhibiting activity of kinases such as c-Src, as well as Janus-activated kinase-1 and -2 (JAK 1/2). STAT3 regulation by CEL controls expression of genes that play a pivotal role in cell proliferation, survival and angiogenesis. CEL's anti-proliferative role has been found to be associated with its pro-apoptotic attribute. Multiple pathways have been reported wherein CEL treatment induced pro-apoptotic signals. For example, inhibition of AKT and mTOR kinases induced apoptosis in glioma cells or a G2/M or G1 phase arrest induced apoptosis in AGS and YCC-2 cells. AMPK-induced p53 and PLK-2 pathways have also been reported to be involved in CEL-induced anti-tumor effects in MCF-7 cells. These diverse mechanisms underscore CEL's potential to target various oncogenic pathways across different cancer types.

Advanced cancers have high pathogenesis due to metastasis or tumor invasion and the prerequisites for tumor invasion are the acute changes in cellular attributes such as cellular adhesion and migration. CEL inhibits TGF-β1-induced epithelial-mesenchymal transition (EMT) by inhibiting snail and regulating E-cadherin expression in lung cancer cells 6. Most importantly, CEL's direct effect on MMP3/MMP7 through PI3K/AKT signaling pathway inhibited migratory and invasive capabilities of osteosarcoma U-2OS cells 7. EMT is also regulated by transcription factors such as heat shock protein 90/transcription factors (HSP90/TFs) and CEL has been reported to affect the interaction of these TFs with genes such as TWIST1 that have critical control over EMT. There is ample evidence supported by robust preclinical studies that indicate CEL has anti-cancerous properties that can be harnessed with multi-modal approaches.

Despite its therapeutic promise, the clinical use of CEL has been stalled by unfavorable solubility, low oral bioavailability, and potential systemic toxicity. To address these limitations, exosomes obtained from milk/colostrum were utilized as a vehicle for the oral delivery of CEL. Exosomes (Figure 1A) are a subclass of extracellular vesicles (EVs), typically 30-150 nm in size, derived from endosomal compartments secreted by virtually all cell types and present in biological fluids, including milk 8-10. In our previous studies, milk-derived exosomes showed lack of toxicity in immune organs such as bone marrow and spleen 11, making them a safe and biocompatible carrier for therapeutic agents. Exosomes represent an attractive drug delivery platform because of their nanoscale size, ability to carry both lipophilic and hydrophilic agents, capacity to protect and stabilize therapeutic cargo, and low toxicity profile.

Exosomes derived from bovine colostrum exhibit high expression of CD47, a transmembrane protein that engages signal regulatory protein-α (SIRPα) on macrophages to deliver a "don't eat me" signal, thereby avoiding phagocytic clearance 12, 13. This property, which is absent in many synthetic nanoparticles 14, 15, enhances the circulation time and tumor accumulation of exosomal formulations. Our own characterization of colostrum-derived exosomes confirms elevated CD47 expression, supporting their potential as long-circulating delivery vehicles.

Folic acid (FA), a tumor targeting ligand, can be conjugated to exosomes to enhance the tumor targetability of ExoCEL. Folate receptor alpha (FRα) is minimally expressed in most normal tissues but is overexpressed in a majority of NSCLC subtypes 16. Studies using tissue microarrays from over 180 patients showed FRα overexpression in 72% of lung adenocarcinomas and 51% of squamous cell carcinomas 17. Additional studies confirm this pattern, with 60% of adenocarcinomas and 33% of squamous cell carcinomas showing high FRα expression 18. Furthermore, folate receptor beta (FRβ) is highly expressed in tumor-associated macrophages (TAMs), which promote tumor progression in both histologic subtypes, further expanding the potential targeting window 18. Importantly, FA is not retained by the kidneys, and FR-targeted agents have shown no significant toxicity in rodent or human studies 19-21.

Together, these findings provide robust justification for the development of a folate-targeted, exosome-based oral delivery system for CEL. This strategy holds promise for improving the therapeutic index of CEL in NSCLC by enhancing bioavailability, increasing tumor specificity, reducing systemic toxicity, and overcoming drug resistance. In this study, we formulated and characterized CEL-loaded exosomes (ExoCEL) and their FA-functionalized counterpart (FA-ExoCEL), assessed their anti-cancer activity in both drug-sensitive and drug-resistant NSCLC cell lines, and evaluated their effects on cell proliferation, migration, EMT, and apoptosis. Finally, we investigated the *in vivo* therapeutic potential of orally administered FA-ExoCEL in an orthotopic lung tumor model. This approach aims to address key challenges in lung cancer therapy by improving oral bioavailability, enabling tumor-specific delivery, and overcoming drug resistance through a biocompatible and patient-friendly nanoplatform.

## Methods

### Chemicals and reagents

CEL (99.6% purity) and PAC were purchased from Chromadex (Irvine, CA) and LC Labs (Woburn, MA), respectively. Protein Assay Kit (BCA) and FA were procured from Thermo Fisher (Waltham, MA) and Sigma-Aldrich, respectively. Potassium salt of D-luciferin (xenoLight) was procured from PerkinElmer (Waltham, MA). Reagents not specifically mentioned above were of analytical quality.

### Exosomes isolation

Exosomes were isolated using colostrum powder obtained from Immunodynamics, Inc. (Fennimore, WI) 22, 23. Briefly, exosomes were isolated from a 5% (w/v) aqueous suspension of colostrum powder through a series of differential centrifugation steps (13,000 x g for 30 min; 65,000 x g for 60 min; and then 135,000 x g for 2 h). The final exosome pellet is subjected to filtration through 300K molecular weight cut off (MWCO) filter prior to use in studies. This filtration removes any free immunoglobulins and other non-vesicular proteins, which has been verified by western blot analysis and measurement of the size of components of the flow through. Purified exosomes were resuspended in PBS (pH 7.4), sterilized by passage through a 0.22-µm filter, and their yield determined by BCA protein quantification before storage at concentrations ≤ 6 mg/mL at -80 °C.

### Exosome characterization

Particle size, PDI and zeta potential were assessed in triplicates using a Zetasizer (Malvern, Worcestershire, UK). The exosome size was confirmed by atomic force microscopy (AFM) as previously reported 23. Exosomes were analyzed for exosomal surface protein markers (Alix and CD81) as well as non-exosomal markers (Calnexin and GM130) by western blot as described 24.

### FA-functionalization of exosomes for tumor-targeting

Folic acid (FA) was conjugated to the exosome surface. First, activated FA was prepared by standard EDC (1-ethyl-3-(3-dimethylaminopropyl) carbodiimide hydrochloride) and NHS (N-hydroxysuccinimide) chemistry. FA was covalently linked to exosomal proteins via an activated folate approach to ensure *in vivo* stability. Free FA was removed by repeated ultrafiltration using 300 kDa MWCO filters. The extent of FA functionalization was controlled by adjusting the FA concentration. To determine the loading, FA was released under alkaline conditions (NaOH), and exosomes were recovered and measured spectrophotometrically. In three preparations we got FA loading of 5.8 ± 0.6%.

### Loading of CEL on exosomes

CEL loading was achieved using our previously reported method 23, with minor modifications including the use of colostrum exosomes, and an increased exosome-to-CEL ratio of 1:3. The CEL loaded exosomes were collected by ultracentrifugation, pellet was washed twice with PBS to make sure there is no residual ethanol. The exosome were suspended in PBS, filter sterilized and the ExoCEL and FA-ExoCEL formulations were stored at -80 °C.

### Determination of CEL loading

CEL loading was determined by UPLC quantification of the drug and BCA measurement of exosomal protein as previously reported 23. A 50 µL aliquot of ExoCEL was mixed with acetonitrile (950 µL), enabling CEL extraction while simultaneously precipitating exosomal proteins. Following centrifugation (10,000 × g, 10 min), the CEL-containing supernatant was subjected to UPLC analysis (5 µL injection), and the recovered protein pellet was resuspended in water for quantification by the BCA assay.

### Labeling of exosomes with cyanine 7

Exo and FA-Exo were covalently labeled with Cyanine-7 (Cy7) using amide chemistry, as described for FA-functionalization. Briefly, FA-Exo (10 mg/mL) were mixed with 100 µL of 1 M NaHCO₃ (pH 8.4), followed by the addition of Cy7 (100 µg/mL), and gently shaken in the dark (1 h). Unbound Cy7 was removed by repeated ultrafiltration using a 300 kDa MWCO filter. The Cy7-labeled exosomes were resuspended in PBS and stored at -80 °C until further use.

### UPLC analysis

UPLC was performed using a 2.2 μm particle size containing RP Shimadzu column (150 mm length × 3.0 mm diameter) to quantify CEL content in the ExoCEL formulation. The mobile phase in a gradient (0.7 mL/min) consisted of 1% citric acid (pH 2.5) and acetonitrile (ACN). The ACN was increased from 5% to 60% between 1.3 and 5.1 min, then to 80% at 7.7 min, followed by a rise to 100% at 10 min. This composition was held until 10.9 min and then returned to 5% by 12 min. CEL was detected at 430 nm using a photodiode array UV detector (PDA-UV), and CEL conc. were determined using calibration curve generated with reference CEL standards.

### Mechanism of drug loading

The intrinsic fluorescence of exosomal proteins arises mainly from aromatic amino acids and leveraged to assess exosomes fluorescence quenching upon CEL interaction, indicative of hydrophobic interactions, as previously described 25. Exosomes and ExoCEL formulation were evaluated using a SpectraMax spectrofluorometer, with wavelengths set at 280 nm for excitation and 320 nm for emission, respectively. A concentration-dependent decrease in fluorescence intensity was observed in ExoCEL samples, suggesting that strong hydrophobic interactions between CEL and exosomal surface proteins play a key role in drug loading.

### Proteinase K-based assessment of surface-exposed IgGs on exosomes

Proteinase K digestion was used to distinguish surface-associated exosomal proteins from lumen-protected cargo on exosomes. Exosomes and ExoCEL were treated for 30 min with Proteinase K (5-80 µg/mL) at 37 °C to selectively digest only the surface-exposed proteins. For drug localization studies, exosomal formulations were passed through MWCO filters following enzymatic treatment and 3X washed with PBS to remove digested proteins and released drug. The retained exosomal fractions were then analyzed for residual CEL content. The percentage loss of CEL following Proteinase K treatment was considered representative of surface-bound drug, while the remaining fraction was attributed to lumen-encapsulated CEL.

To assess presence of IgGs on exosome surface, native exosomes were treated with Proteinase K under identical conditions and analyzed by immunoblotting for IgG and CD81, both surface-exposed exosomal proteins.

### *In vitro* release study

The *in vitro* release profile of CEL from FA-ExoCEL was evaluated using four different release media to simulate physiologically relevant gastrointestinal and systemic conditions. Fasted state simulated gastric fluid (FaSSGF, pH 1.6), fasted state simulated intestinal fluid (FaSSIF, pH 6.5), and fed state simulated intestinal fluid (FeSSIF, pH 5.8) were purchased from Biorelevant (London, United Kingdom), while PBS (pH 7.4) was used to represent physiological conditions. FA-ExoCEL (1 mL) was filled in the dialysis tubes (Tube-O-DIALYZER™, G Biosciences) and incubated for 4 h in 25 mL) were collected at different time intervals and the remaining CEL content was measured spectrophotometrically.

### Cell lines and culture conditions

Human NSCLC cell lines H1299 and A549, normal human bronchial epithelial cells (BEAS-2B) and epithelial mammary cell line (MCF 10A) were obtained from ATCC (Manassas, VA, USA), while the taxol-resistant A549TR cell line was provided by Dr. Bruce Zetter (Harvard Medical School, Boston, MA). All these cell lines were maintained as described previously 23. Bioware® Brite A549 Red-FLuc cells (PerkinElmer, USA), engineered for *in vivo* imaging, were cultured in RPMI-1640 medium supplemented with 10% FBS. To maintain luciferase activity and reduce selection pressure, no antibiotics were added to the culture media. These cells were also incubated at 37 °C with 5% CO₂ under standard conditions.

### *In vitro* antiproliferative activity and colony-forming assay

The antiproliferative and long-term growth-inhibitory effects of CEL and ExoCEL were determined using MTT-based cell viability and colony-forming assays, respectively, as previously reported 22, 23, 26. Briefly, A549, A549-TR, H1299, BEAS-2B, and MCF10A cells were plated in 96-well plates and treated with free CEL, Exo, or ExoCEL for 24-72 h, followed by assessment of cell viability. For colony forming analysis, A549 and H1299 cells were treated with CEL or ExoCEL for 24 h, and cultured in drug-free medium to allow colony formation, and colonies were subsequently fixed, stained, and quantified 26.

### Wound healing assay

Wound healing (scratch) assay was performed for cell migration assessment. A549 and H1299 cells (1 × 10⁴ cells/chamber) were plated into a two-well culture-insert system (Ibidi, Gräfelfing, Germany) and allowed to attach for 24 h. After incubation, a uniform wound gap was created by removing the inserts with sterile forceps. Cells were washed to remove debris and treated with media containing varying concentrations of CEL in the presence and absence of TGF-β (5 ng/mL). Wound closure was monitored at 0, 8, 24, and 48 h using phase-contrast microscopy. Images were captured and analyzed quantitatively using Wimasis image analysis software and Image J software using the Wound Healing Size Tool 27.

### Cellular uptake of exosomes

Cellular uptake of exosomes was assessed by fluorescent labeling and imaging-based quantification. Exosomes were labeled with cyanine 7 (Cy7), and lung cancer A549 and H1299 cells were seeded in 24-well clear-bottom plates at a density of 7.5 × 10⁴ cells per well. After overnight attachment, cells were treated with free Cy7 or Cy7-labeled exosomes (Exo-Cy7) at an equivalent fluorescence intensity (5000 RFU/mL) and incubated for 4 h. Four hours incubation was selected based on our prior study 28. Following incubation, the medium was removed, cells were gently washed with PBS, and fluorescence images were acquired using the Lionheart-FX automated microscope. Cellular fluorescence intensity was quantified using ImageJ software. All treatments were performed in triplicate.

### Transwell migration assay

Cell migration was assessed using 8 µm transwell pore inserts as previously described 26. CEL 1 h treated cells were collected and counted. Cells (4 × 10⁴ in 200 µL) were seeded in DMEM without FBS supplement into the upper chamber. The lower compartment contained 600 µL FBS supplemented DMEM. Non-migrated cells were removed from the upper surface after 24 h, and migrated cells in the lower side were fixed with paraformaldehyde (4%), permeabilized with methanol, and stained with toluidine blue (0.2%). Migrating cells were quantified in five randomly selected microscopic fields and reported as representative images and mean ± SD.

### RNA sequencing (RNA-Seq) analysis

Total RNA was isolated from Veh and CEL (1 µM) treated A549 cells using the QIAGEN QIAwave RNA Plus Mini Kit (Catalog #74634, Qiagen, Hilden, Germany). Total RNA quantity and quality were evaluated by a NanoDrop, followed by submission for poly(A) RNA-seq at Novogene. RNA integrity was evaluated with the Agilent Bioanalyzer 2100 system. High-quality RNA samples underwent poly-A selection and were used for library preparation with ABclonal's Fast RNA-seq Lib Prep Kit V2 (Catalog #RK20306). After mRNA purification and fragmentation, first-strand cDNA was generated with random hexamer primers, and second-strand synthesis was subsequently performed using either dTTP to produce non-strand-specific libraries or dUTP to generate strand-specific libraries. The resulting libraries were quantified using a Qubit fluorometer and real-time PCR. Library fragment size distribution and quality were assessed using the Agilent Bioanalyzer 2100 system. Final libraries were pooled and sequenced on the Illumina NovaSeq X Plus platform using paired-end sequencing.

Quality control (QC) and trimming of raw sequencing data were performed using in-house Perl scripts developed by Novogene. Alignment of high-quality reads to the human reference genome (GRCh38/hg38) was performed using HISAT2 (v2.5) 29. Differential gene expression analysis was conducted with the DESeq2 R package (v1.20.0), using raw gene count data as input 30. DESeq2 applies a negative binomial distribution model and performs internal normalization to identify differentially expressed genes. Statistical significance was determined according to DESeq2 guidelines, with P-values adjusted for multiple testing using the Benjamini-Hochberg procedure (adjusted p ≤ 0.05). Functional enrichment analysis was carried out using STRING (v12.0) 31.

H1299 cells naturally exhibit a mesenchymal phenotype and are more suitable than A549 cells for EMT studies. Thus, to directly compare the effects of free CEL and its exosomal formulation (ExoCEL) on TGF-β-induced EMT, RNA-seq analysis was performed. Briefly, H1299 cells were pretreated with TGF-β (5 ng/mL) for 2 h to induce EMT, then the medium was replaced with media containing CEL or ExoCEL (0.5 µM) at equivalent CEL concentration. Following treatment, cells were collected by centrifugation, resuspended in DNA/RNA Shield (Zymo Research, USA) and sent for further analysis to Plasmidsaurus (Louisville, KY) for RNA isolation, library preparation, and transcriptomic analysis.

### Protein extraction and Western blot analysis

To examine the molecular effects of CEL and its exosomal formulation, A549 and H1299 cells were treated with different concentrations of CEL and ExoCEL and TGF-β (5 ng/mL) for 48 h. Proteins were extracted using RIPA buffer with protease inhibitors, quantified by BCA assay. Proteins were separated by SDS-PAGE, and transferred to PVDF membranes for immunoblotting 22. Blots were probed with target specific primary antibodies, and band intensities were quantified relative to β-actin using ImageJ software (NIH, Bethesda, MD).

### qRT-PCR for target gene expression

Lung cancer H1299 and A549 cells were treated with vehicle or varying concentrations of CEL, and total RNA was isolated 24 h post-treatment using TRIzol reagent (Invitrogen, Carlsbad, CA). Quantitative real-time PCR was performed on 200 ng of RNA using a One-Step SYBR Green qRT-PCR Kit. (Quanta Biosciences, Gaithersburg, MD), which enables simultaneous cDNA synthesis and amplification as previously described 32. Sequences of the primer used in this study are provided in Table S2. Relative transcript levels were calculated by ^ΔΔ^Ct analysis using β-actin for normalization. 33. Fold changes in gene expression were expressed as 2^ΔΔCt^. All reactions were performed in triplicate.

### Biodistribution of exosomal formulations in lung-tumor bearing mice

Female C57BL/6 mice bearing orthotopic lung tumors were randomized into four groups and a single oral dose of free Cy7, Exo-Cy7, or FA-Exo-Cy7 (n = 3 per group) was administered. In each treatment, Cy7 signals were kept constant at 100,000 fluorescent units. One untreated mouse served as a control. Mice were fasted for 4 h prior to dosing, and fasting was maintained for 12 h post-administration. At 12 h after treatment, animals were euthanized, and major organs including tumor-bearing lung tissue were harvested and imaged *ex vivo* using the Odyssey LI-COR CLx system. Fluorescence intensities from each tissue were quantified for biodistribution analysis.

To further confirm folate receptor-mediated tumor targeting, an additional group of tumor-bearing mice was pretreated with free FA prior to administration of FA-Exo-Cy7. Briefly, mice received two doses of FA (10 mg/kg per dose) orally at defined time intervals before administration of FA-Exo-Cy7. All other experimental conditions, including Cy7 dose, fasting schedule, imaging time, and tissue collection remained identical to those described above. At 12 h, organs were harvested and analyzed *ex vivo* for Cy7 levels to assess the effect of folate receptor saturation on FA-Exo-Cy7 tumor accumulation.

### Gastrointestinal uptake of FA-Exo following oral administration

To evaluate the mechanism of GI absorption of FA-Exo following oral administration, we hypothesized that uptake is mediated by neonatal Fc receptors (FcRn), which are known to bind bovine immunoglobulins (Igs) with high affinity 34. To test this, female C57BL/6 mice (n = 3 per group) were randomized into two groups and treated with a single dose of Cy7-labeled FA-Exo or Cy7-labeled FA-Exo with Ig pretreatment (100 mg/kg; Igs + Cy7-FA-Exo). Each animal received 50,000 fluorescent units of Cy7-FA-Exo. Mice were fasted for 3 h prior to Ig administration and received Cy7-FA-Exo 1 h later; fasting was continued for 12 h post-dosing, with *ad libitum* access to sterile water. Blood samples (50-60 µL) were collected from the jugular vein at 0, 2, 6, and 24 h. At each time point, serum was separated by centrifuging the blood at 300 rpm and 5 µl from every animal (in triplicate) was spotted on a cover slip and imaged using the Odyssey LI-COR CLx system to quantify fluorescence in each sample.

### Assessment of intact exosomes in serum following oral administration

To evaluate whether orally administered exosomes can cross the gastrointestinal tract and be detected in the circulation in an intact form, serum exosomes were analyzed following oral dosing with bovine exosomes in a pilot study. Athymic nude mice (n = 2-3) received vehicle or colostrum exosomes (5 mg per mouse) orally. Four hours after administration, mice were euthanized, and blood was collected by cardiac puncture. Serum was separated by centrifugation at 2,500 rpm for 15 min. The exosomes were then isolated from serum by incubating with ExoQuick-TC reagent (System Biosciences, Palo Alto, CA). The recovered vesicles were quantified for total protein content and analyzed by western blotting for bovine specific exosomal markers CD81 and CD63.

### Orthotopic lung tumor model and *in vivo* treatment

All animal procedures were conducted in accordance with protocols approved by the Institutional Animal Care and Use Committee. For the orthotopic lung tumor model, female NOD-SCID mice were inoculated with 2 × 10⁶ Bioware® Brite A549 Red-FLuc cells suspended in 50 µL of a 1:1 (v/v) mixture of Matrigel and serum-free medium. Injections were performed intrathoracically using 30-gauge needles, as previously described 35, 36. Tumor development was monitored via bioluminescence imaging, and once the luminescence signal reached approximately 6 × 10⁶ photons (typically by day 10 post-inoculation), mice were randomized into treatment groups (n = 10 per group). Mice received either vehicle (PBS) or FA-functionalized CEL-loaded exosomes (FA-ExoCEL), administered orally at a dose of 8 mg/kg, three times per week. We compared the efficacy of FA-ExoCEL in this study with CEL and ExoCEL from our previously published study 23. Throughout the study, body weight, food intake, and general health status were monitored weekly.

Tumor growth was assessed biweekly by measuring luciferase activity using the AMI-HTX (SII, Tucson, AZ). Fifteen min prior to imaging, mice were injected intraperitoneally with D-luciferin (120 mg/kg). At the end of the study, animals were euthanized, and tissues including lungs, liver, and tumors were collected and stored for molecular analysis.

### Comparative *in vivo* evaluation of CEL, ExoCEL, and FA-ExoCEL

To directly assess whether exosomal loading and FA-functionalization enhance the oral antitumor efficacy of CEL, an additional orthotopic lung tumor study was performed in which free CEL, ExoCEL, and FA-ExoCEL were evaluated side-by-side within the same experiment. Female athymic nude mice bearing orthotopic A549-Red-FLuc lung tumors were assigned to four groups (n = 10 per group) and treated orally with CEL, ExoCEL, or FA-ExoCEL. The dose of CEL in all regimen and other experimental conditions were same as described in above study. Tumor progression was assessed by bioluminescence imaging, and body weight, food intake, and overall health were monitored throughout the study. At study termination, lungs and major organs were harvested for biomarker analysis and histological analyses. The tumor samples were used to analyze molecular markers by western blot analysis.

### Assessment of systemic toxicity

For systemic toxicity, female wild-type C57BL/6 mice (5-6 weeks of age; n=5) were orally treated three times a week with vehicle or FA-ExoCEL (8 mg/kg) for four weeks. Following euthanasia, blood and serum were collected and analyzed for hematological, biochemical, and electrolyte parameters as previously described 23.

### Chemosensitization of paclitaxel-resistant cells by CEL

To evaluate the antiproliferative activity of CEL against PAC-resistant lung cancer cells, MTT assays were performed using A549 and taxol-resistant A549TR cell lines. After seeding at 3 × 10³ cells per well and overnight attachment, cells were treated with varying concentrations of CEL, ExoCEL, PAC, or combination of PAC and CEL. After 72 h of treatment, the antiproliferative activity was determined by MTT assay. Dose-response curves for the CEL and PAC combination were generated using cell viability data using CalcuSyn software version 2.0 (Biosoft), based on the median-effect method.

### Statistical analysis

All statistical analyses were conducted using GraphPad Prism (La Jolla, CA, USA). Data are expressed as mean ± SD or mean ± SEM, as indicated. Differences between groups *in vitro* studies were evaluated using unpaired Student's *t*-test. For tumor xenograft study, two-way ANOVA followed by Bonferroni's multiple correction test was conducted. Data is shown for n = 8 animals per group. A p value of < 0.05 was considered statistically significant.

## Results

### Bovine colostrum is a scalable source of exosomes

Previously we have shown the isolation of exosomes from bovine milk 23. Particle size and the presence of key surface protein markers (CD63, CD81, Alix, and Tsg101) confirmed that these milk-derived particles were exosomes 24. In this study, the exosomes were isolated from colostrum powder through rehydration, followed by a differential centrifugation procedure. Particle size distribution and surface charge analysis demonstrated that the isolated exosomes averaged 66 ± 7.7 nm in diameter, with a PDI of 0.265 ± 0.02 and a zeta potential of -15.53 ± 0.71 mV. AFM yielded similar size measurements (Figure 1C and Figure S1). The isolated exosomes were positive for the exosomal markers CD81 and Alix, while the endoplasmic reticulum and Golgi markers calnexin and GM130 were not detected (Figure 1D), confirming the purity of the exosomal preparation. Exosomal quality was consistent across different preparations based on protein marker expression. Typically, 100 g of colostrum powder yielded 1.03 ± 0.06 g of exosomal protein, while 1 L of liquid colostrum (equivalent to ~220 g powder) provided 2.27 g of exosomal protein and 3.9 × 10¹⁵ particles 37. Exosomes suspended in PBS and stored at -80°C remained stable in terms of size and biological activity for several months.

### Loading of CEL onto exosomes (Exo) and FA-Exo

CEL loading was achieved by a short incubation of Exo in PBS with CEL in 10% ethanol 23. The average particle size of exosomes after loading CEL was insignificantly increased to 83 ± 5.1 nm, PDI of 0.288 ± 0.01 and ZP of -14.9 ± 2.16 mV (Figure 1B and E; Table S1). To analyze the CEL loading, ExoCEL was mixed with acetonitrile which resulted in CEL extraction and precipitation of the Exo protein. UPLC and BCA methods were used to measure the CEL and exosomal proteins, respectively. A drug load of 22 ± 3% was achieved for CEL. The CEL loading on FA-Exo did not affect the particle size (91 ± 8.3 nm), PDI (0.278 ± 0.02) and ZP (-16.7 ± 2.23) (Table S1).

### Mechanisms of drug loading in exosomes

Exosomes possess a range of transmembrane proteins that may serve as binding sites for lipophilic drugs through hydrophobic interactions. To investigate whether hydrophobic interactions contribute to drug loading onto exosomes, we measured the intrinsic fluorescence of unloaded exosomes (Exo) and ExoCEL. Our data (Figure 1F) revealed ~80% reduction in fluorescence intensity after drug loading, supporting the hypothesis that strong hydrophobic interactions between CEL and exosomal surface proteins play a major role in drug incorporation. Nonetheless, we cannot exclude the possibility that a CEL fraction is also embedded within the lipid bilayer of exosome or luminally.

To better understand where CEL is located on or within exosomes, ExoCEL was treated with increasing concentrations of Proteinase K to remove surface-exposed proteins. We found concentration-dependent reduction in CEL levels (Figure 1G) with proteinase K treatment, indicating that a significant percentage of CEL is bound to exosomal surface proteins. CEL was not completely removed by protease treatment, suggesting that some of the drug is likely embedded within the exosomal lipid membrane or present inside the lumen.

### *In vitro* release study

*In vitro* release studies provided additional understanding into CEL localization within FA-ExoCEL. Exosomal formulation showed a time-dependent release across all tested media (Figure 1H). In intestinal-simulating media, including FeSSIF (pH 5.8) and FaSSIF (pH 6.5), FA-ExoCEL showed a gradual increase in CEL release, reaching approximately 40-50% by 4 h. In contrast, release under gastric conditions (FaSSGF, pH 1.6) remained minimal over the same time period, indicating good stability in acidic environments. When evaluated in PBS (pH 7.4), ExoCEL exhibited a sustained and higher extent of release, reaching ~40% over 24 h.

### CEL inhibits the growth of NSCLC cells *in vitro*

To determine if CEL is effective against lung cancer, H1299 and A549 cells were used. MTT data show time- and dose-dependent anti-proliferative activity of CEL against H1299 and A549 with IC₅₀ 1.4 and 1.8 µM, respectively 23. CEL also exhibited comparable antiproliferative activity against drug-resistant A549TR cells. In contrast, CEL showed minimal cytotoxicity in normal bronchial epithelial (Beas2B) and mammary epithelial (MCF10A) cells, with over five-fold higher IC₅₀ values, indicating selective activity against cancer cells (Figure 1I). Thus, unlike most anticancer drugs, CEL selectively targets tumor cells and causes no obvious damage to normal hematopoietic cells 38.

### CEL attenuates lung cancer cell colony-forming ability

**Figure 2:**
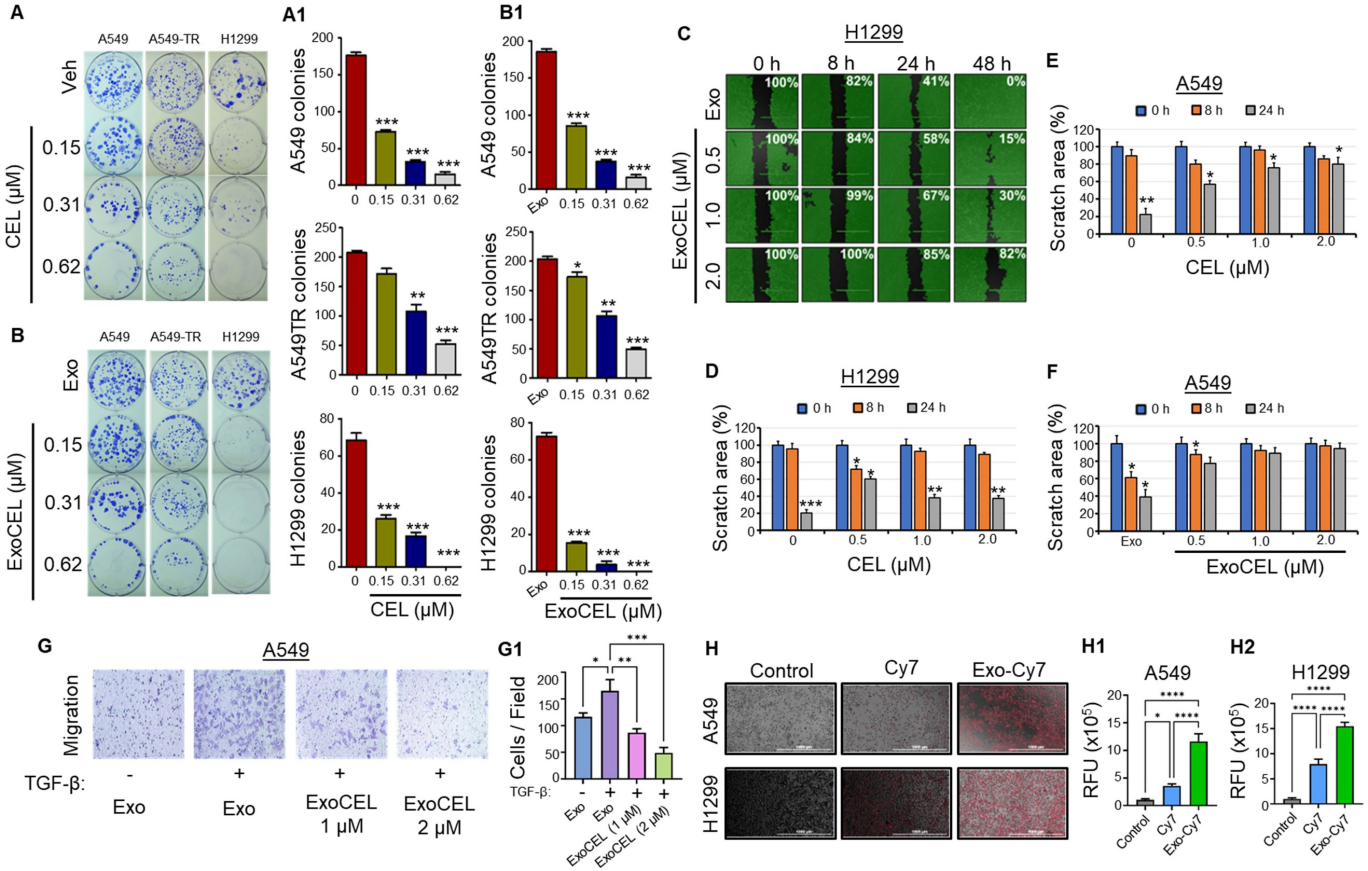
** CEL and ExoCEL inhibit colony formation and cell migration in lung cancer cells.** A549, A549-TR (taxol-resistant), and H1299 cells were treated with increasing concentrations of CEL or its exosomal formulation (ExoCEL) for 24 h, followed by drug-free culture for 10 d. Representative images **(A, B)** and quantification (A1, B1) show a dose-dependent reduction in colony formation across all cell lines. ExoCEL was particularly effective in H1299 and A549-TR cells. Data represent mean ± SD (n = 3). **ExoCEL inhibits cell migration in A549 and H1299 cells (C-G).** A549 cells were seeded in 6-well plates with a silicon culture insert to create a uniform wound. After 24 h, inserts were removed, and cells were washed three times before treatment with different concentrations of ExoCEL (0.5, 1.0, and 2.0 µM). Images were captured at 0, 8, 24, and 48 h after the treatment. Percent scratch area coverage is indicated in each panel relative to the initial wound at 0 h. (A and A1) Quantitative analysis of scratch area over time, showing reduced migration with increasing ExoCEL concentrations. (C-F) Comparative analysis of wound closure in H1299 (C, D) and A549 (E, F) cells treated with free CEL and ExoCEL. Both CEL and ExoCEL inhibited migration, with ExoCEL showing stronger suppression, especially at higher doses. Representative images (G) showing the effect of ExoCEL on transwell migration of A549 cells, and bar diagram shows migratory cells per field (G1). Data indicates a consistent delay in wound closure, supporting the role of ExoCEL in impairing cell motility and potentially limiting metastasis. **(H)** Representative fluorescence microscopy images showing cellular uptake of free Cy7 and Exo-Cy7 by A549 and H1299 cells after 4 h of incubation. Quantitative analysis of intracellular fluorescence showed significantly enhanced uptake of Exo-Cy7 compared with free Cy7 in in A549 cells (H1) and H1299 Cells (H2). Statistical analysis was performed using one-way repeated measures ANOVA followed by Dunnett's multiple comparisons test. *p < 0.05, **p < 0.01, ***p < 0.001 vs. untreated control.

To confirm antiproliferative activity, the effects of free CEL and exosome-encapsulated CEL on colony formation were assessed in both drug-responsive (A549, H1299) and resistant (A549-TR) lung cancer cells (Figure 2A & 2B). Both CEL and ExoCEL inhibited colony formation dose-dependently. Free CEL and ExoCEL reduced A549 colony formation by 59% and 54%, respectively, at 0.15 µM (p < 0.001) and by 91-94% at 0.62 µM (p < 0.001). In A549-TR cells, lower doses (0.15 µM) had minimal effects (17% and 15% inhibition), whereas higher doses (0.62 µM) significantly reduced colonies by 73-76% (p < 0.001) (Figure 2A & 2A1). The effects on A549 and A549-TR were comparable, with no substantial advantage from the Exo formulation. However, ExoCEL was more effective in H1299 cells, inhibiting colony formation by 79-95% at 0.15 and 0.31 µM, compared to 62-76% with CEL (p < 0.001). At 0.62 µM, both CEL and ExoCEL completely suppressed H1299 colony formation (Figure 2B & B1).

### ExoCEL inhibits cell migration

The effect of ExoCEL on cell migration was evaluated using a scratch wound healing assay over 48 h and transwell cell migration assay. As shown in the representative micrographs (Figure 2C), treatment with ExoCEL inhibited wound closure dose-dependently. In the untreated Exo control group, the wound area progressively decreased, with nearly complete closure (0% remaining wound area) observed by 48 h. In contrast, ExoCEL-treated cells displayed impaired migratory ability. At 0.5 µM, wound closure was delayed, with 15% of the wound area remaining at 48 h. Increasing the concentration to 1.0 µM resulted in further inhibition, with 30% of the wound area persisting. Notably, the highest concentration of ExoCEL (2.0 µM) maintained 82% of the original wound area at 48 h, indicating strong suppression of cell migration.

Quantitative analysis confirmed that CEL and ExoCEL significantly and dose-dependently inhibited cell motility compared to control in both A549 and H1299 (Figure 2D & 2F). These results suggest that ExoCEL effectively impairs cancer cell migration, may potentially contribute to its anti-metastatic activity. To confirm the CEL's ability to inhibit migration, we conducted a transwell migration assay in the absence and presence of TGF-β. ExoCEL inhibited cell migration significantly through the transwell membrane in a dose-dependent manner (Figure 2G & 2G1).

### Cellular uptake of exosomes

Representative fluorescence microscopy images showed uptake of both free Cy7 and Exo-Cy7 by A549 and H1299 cells after 4 h of incubation (Figure 2H). Quantitative analysis of cellular fluorescence revealed significantly higher uptake of Exo-Cy7 compared with free Cy7 in both cell lines (Figure 2H1 & H2; Figure S2 & S3). Exo-Cy7 increased intracellular fluorescence by ~11.7% in A549 cells and ~16.3% in H1299 cells relative to untreated controls, whereas free Cy7 produced only modest increases of ~3.5% and ~7.9%, respectively. These findings demonstrate that exosomal encapsulation markedly enhances cellular uptake of the fluorescent payload compared with the free dye. As H1299 showed more uptake of exosomes, this could be the plausible reason for higher sensitivity to ExoCEL in colony forming and MTT assays.

### RNA Seq analysis revealed that CEL modulated EMT genes

**Figure 3:**
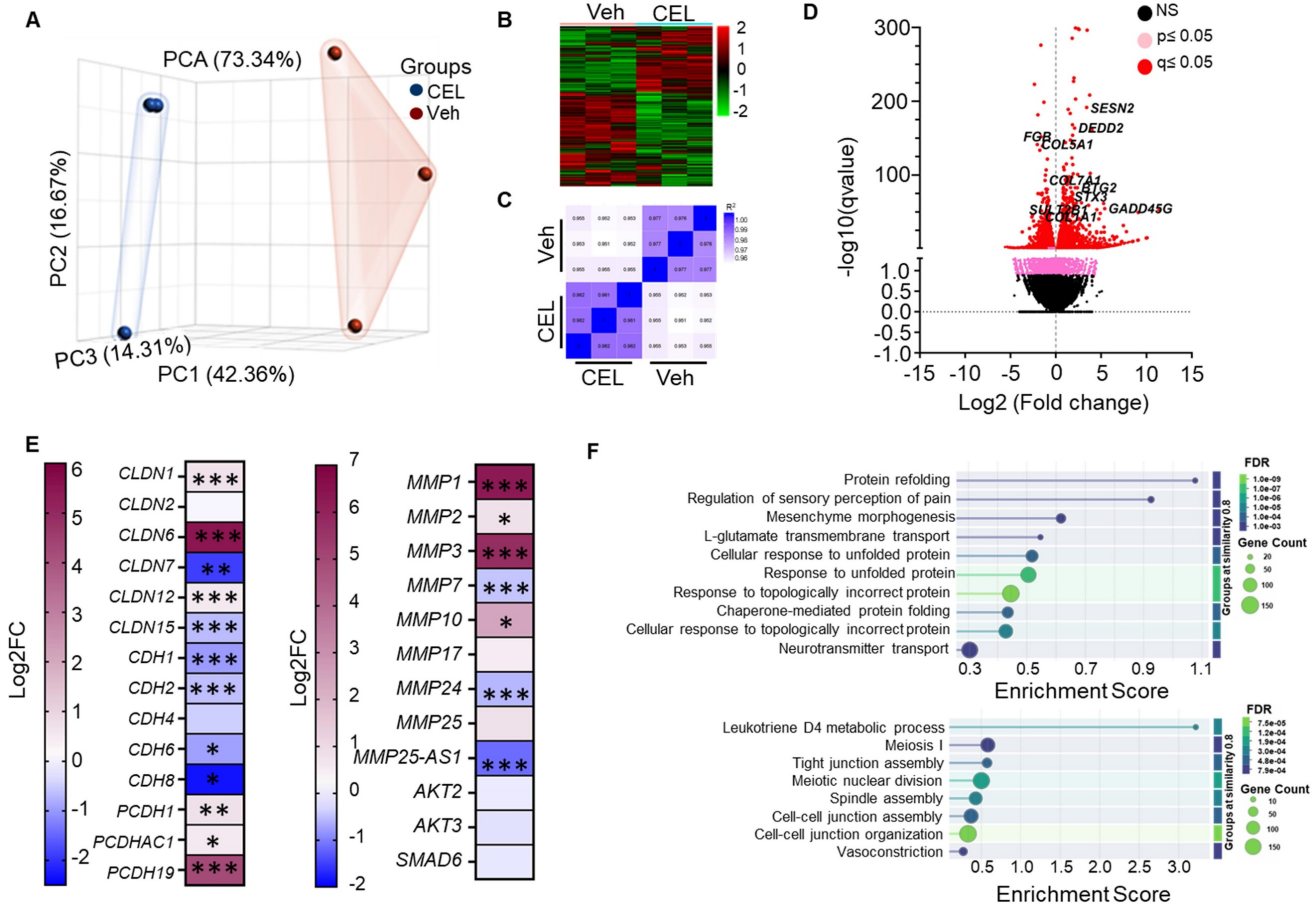
** RNA seq analysis of vehicle and CEL treated A549 cells. (A)** Principal Component Analysis (PCA) of normalized gene counts for vehicle (Veh) and CEL-treated A549 cells where each point represents an individual sample. Color intensity represents the strength of correlation, with darker blue indicating higher correlation values. **(B)** Clustered heatmap depicting differential expression of genes in Veh and CEL. Red denotes upregulation and green denotes downregulation. **(C)** Pearson correlation coefficient heatmap comparing gene expression profiles between Veh and CEL. **(D)** Volcano plot of differentially expressed genes in CEL vs Veh. Novel upregulated and downregulated genes are labelled. **(E)** Heatmaps of the differentially expressed claudins, cadherins and protocadherins (Left Panel) and MMPs (Right Panel). Each cell represents the expression level of the respective gene (rows) for the specific comparison (columns). Colors indicate expression intensity, with blue representing downregulation and purple representing upregulation based on log2FC. *<0.05, **<0.001, ***<0.001. **(F)** STRING Biological Processes (GO Ontology) Enrichments. Up-regulated enriched pathways are on top panel and down-regulated pathways are on bottom panel. Setting: Category: Biological Process (Gene Ontology), Group Terms by: Similarity ≥0.8, Sort Terms by: Enrichment, No of terms: maximum possible, Color Palette: mint-blue, Merge rows by term similarity: Don't merge, Maximum FDR: <0.05, Minimum count in set = 5.

To investigate the molecular mechanisms of CEL, we performed polyA RNA sequencing RNA isolated from CEL-treated lung cancer cells. Principal component analysis (PCA) revealed distinct gene expression profiles for the Vehicle and CEL groups. (Figure 3A). Clustered heatmaps revealed reciprocal gene expression patterns between Vehicle and CEL groups, with distinct upregulation and downregulation profiles across samples. (Figure 3B). The Pearson correlation heatmap demonstrates consistently high intra-group correlations among CEL-treated samples (r ≥ 0.977; dark blue regions) and among Vehicle-treated samples (r ≈ 0.965-0.968). In comparison, inter-group correlations between CEL and Vehicle samples are slightly lower (r ≈ 0.951-0.962; light blue regions), suggesting modest transcriptional divergence induced by CEL treatment. (Figure 3C).

As depicted in the volcano plot, upregulated genes following CEL treatment include *SENP2* (Stress-activated endoplasmic reticulum-nuclease 2), *DEDD2* (Death Effector Domain Containing 2), *BTG2* (B-cell Translocation Gene 2), *STX3* (Syntaxin 3), and *GADD45G* (Growth Arrest and DNA Damage Inducible Gamma). Downregulated genes include *FGB (*Fibrinogen Beta Chain*), COL5A1* (Collagen Type V Alpha 1 Chain)*, COL7A1* (Collagen Type VII Alpha 1 Chain)*, COL1A1* (Collagen Type I Alpha 1 Chain)*,* and *SULT2B1* (Sulfotransferase Family 2B Member 1) (Figure 3D). The dose-dependent downregulation of COL5A1 protein in A549 cells treated with CEL and ExoCEL was further validated using western blot analysis (Figure 5D).

We further examined genes involved in EMT (Figure 3E - left panel). Claudin genes exhibited differential regulation: *CLDN7* and *CLDN15* were downregulated, *CLDN6* was upregulated, while *CLDN1, CLDN2*, and *CLDN12* remained unchanged. Cadherins were broadly suppressed with canonical EMT markers *CDH1* (E-cadherin) and *CDH2* (N-cadherin) both downregulated. Matrix metalloproteinases (MMPs) facilitate EMT, were also modulated. *MMP1* and *MMP3* were significantly upregulated, while *MMP7, MMP24* and *MMP25-AS1* were significantly downregulated. Additionally, CEL treatment downregulated the gene expression of *AKT2, AKT3* and *SMAD6* (Figure 3E - right panel).

Gene Ontology (GO) enrichment analysis revealed the most significantly upregulated and downregulated biological processes (Figure 3F). The top upregulated processes include protein refolding, chaperone-mediated folding, and unfolded protein response processes (Figure 3F - top panel). Conversely, downregulated processes were primarily associated with cell-cell junction formation, maintenance, and cytoskeletal dynamics, indicating disruption in cellular structural integrity (Figure 3F - bottom panel).

### RNA-seq analysis reveals comparable EMT-reversal by CEL and ExoCEL

**Figure 4:**
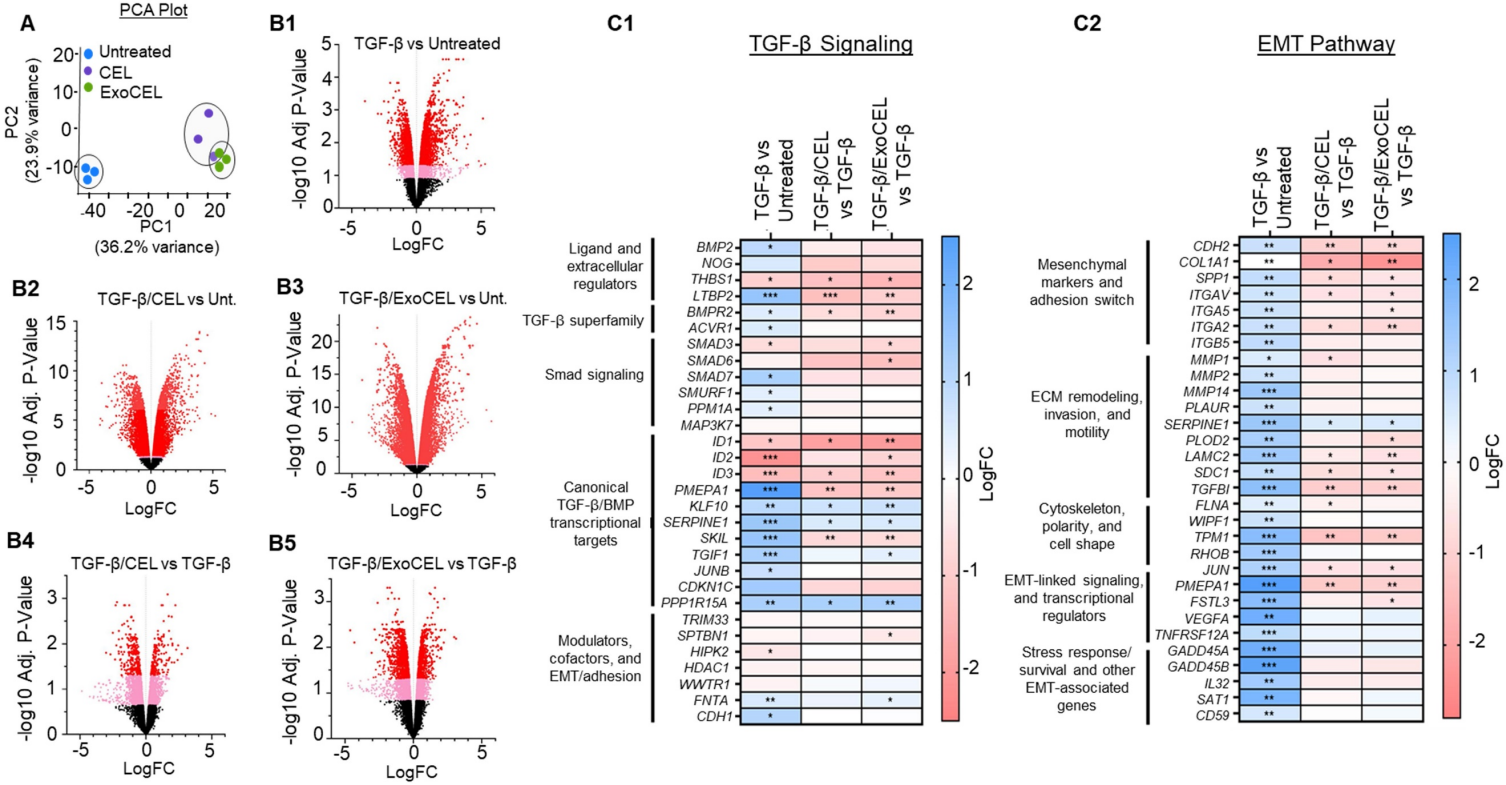
**RNA-seq analysis of reversal of TGF-β-mediated gene modulation by CEL and ExoCEL**. **(A)** Principal component analysis (PCA) of normalized gene expression profiles from untreated cells and TGF-β-treated cells subsequently exposed to CEL or ExoCEL, where each point represents an individual sample. (B1-B5) Volcano plots showing differentially expressed genes across the indicated comparisons. (C1-C2) Clustered heatmaps illustrating differential expression of genes associated with the TGF-β signaling pathway and EMT. First, the genes modulated by TGF-β treatment were identified and then compared with the respective expression patterns in CEL or ExoCEL treated H1299 cells. Blue and red color indicate relative upregulation and downregulation, respectively, based on log fold change (logFC). Statistical significance is indicated as *p* < 0.05, *p* < 0.01, *p* < 0.001.

To determine whether exosomal loading alters the ability of CEL to offset TGF-β-induced EMT, we conducted transcriptomic profiling of H1299 cells treated with free CEL or ExoCEL following EMT induction. Principal component analysis (Figure 4A) showed CEL- and ExoCEL-treated samples clustered closely and distinct to untreated. This shows that exosomal encapsulation does not fundamentally alter the gene regulatory pattern induced by CEL and both treatments elicit similar transcriptional responses.

Volcano plots generated for each comparison further supported this observation, as CEL and ExoCEL produced largely overlapping patterns of differentially expressed genes relative to control (Figure 4B1-B5). As shown in Fig 4C1 and 4C2, TGF-β treatment robustly activated canonical Smad-dependent signaling pathways, as reflected by increased expression of well-established TGF-β target genes, including *PMEPA1, SERPINE1, SKIL, TGIF1, KLF10, JUNB, CDKN1C,* and *PPP1R15A* (Figure 4C1). These changes confirm strong engagement of TGF-β cytostatic and EMT-associated transcriptional programs in lung cancer cells. CEL and ExoCEL effectively modulated expression of the majority of the TGF-β-responsive genes, suggesting their therapeutic utility in counteracting EMT progression. Similarly, TGF-β treatment upregulated pro-EMT genes that regulate mesenchymal adhesion, ECM remodeling, invasion, cytoskeletal dynamics, motility, and EMT-related transcriptional and survival pathways (Figure 4C2). Both CEL and ExoCEL reversed these transcriptional changes, either by significantly suppressing gene expression or by diminishing the degree of TGF-β-induced upregulation. The ability of ExoCEL to mirror the EMT-suppressive effects of free CEL supports its potential as a therapeutically viable formulation for inhibiting EMT and limiting metastatic progression in lung cancer.

### CEL reverses TGF-β-induced EMT in H1299 cells

**Figure 5:**
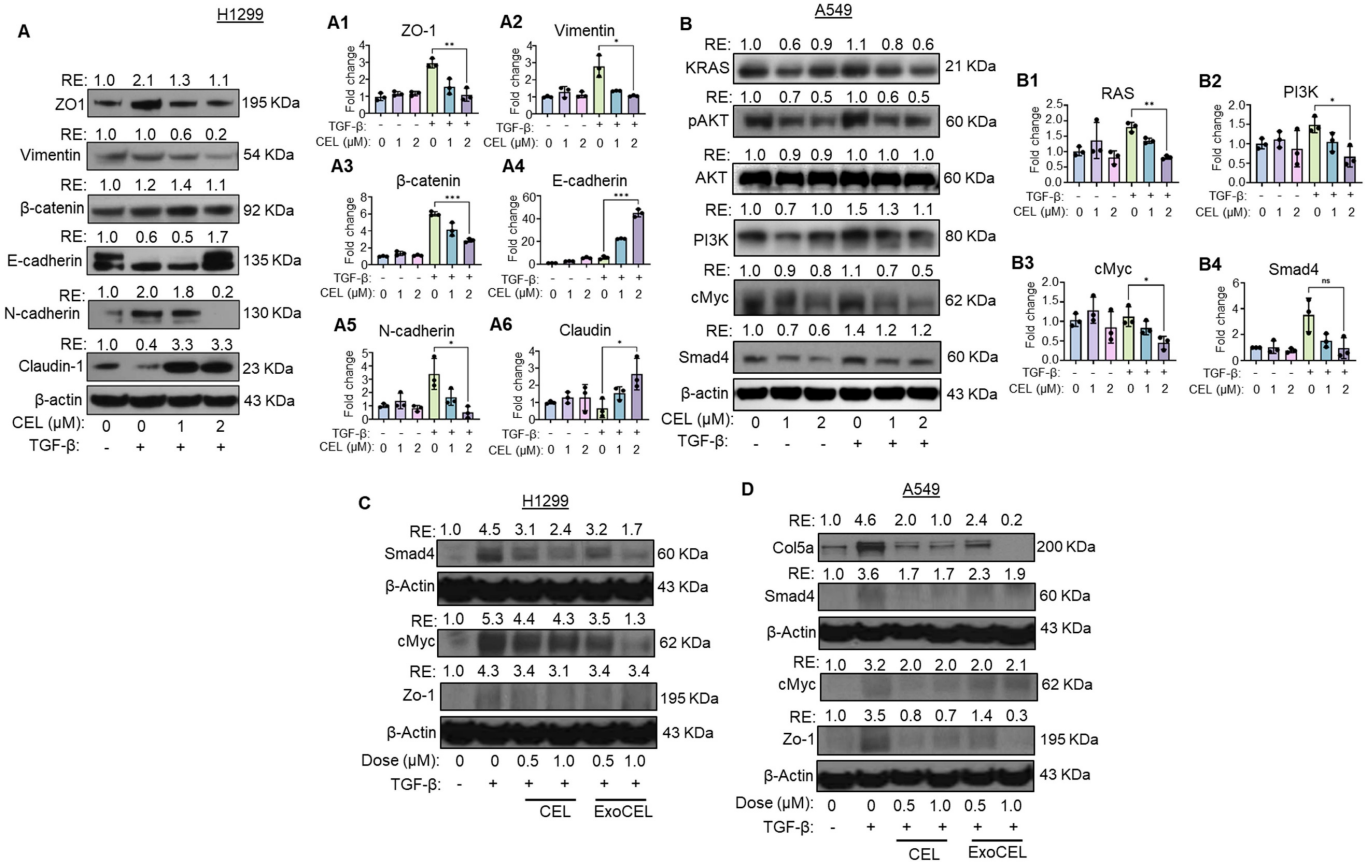
**CEL reverses TGF-β-induced EMT and suppresses oncogenic signaling in lung cancer cells. (A)** H1299 cells were treated with TGF-β (5 ng/mL) for 24 h to induce EMT, followed by treatment with CEL (1 or 2 µM) for an additional 24 h. Western blot analysis revealed that CEL reversed TGF-β-induced mesenchymal changes, as shown by upregulation of epithelial markers (ZO-1, E-cadherin, claudin) and downregulation of mesenchymal markers (vimentin, β-catenin, N-cadherin). **(B)** A549 cells were treated with TGF-β and varying concentrations of CEL for 24 h. CEL treatment resulted in downregulation of KRAS and key oncogenic signaling molecules including phosphorylated AKT (pAKT), PI3K, c-Myc, and Smad4. These findings indicate that CEL effectively inhibits EMT progression and disrupts KRAS-mediated oncogenic and pro-survival pathways in NSCLC cells. H1299 and A549 cells were treated with TGF-β (5 ng/mL, 24 h) to induce EMT, followed by CEL (1 or 2 µM, 24 h). Gene expression analysis in H1299 (A1-A6) and A549 (B1-B4) cells by qRT-PCR for validation of EMT and oncogenic signaling markers confirmed consistent transcript-level changes. **(C, D)** Western blot analysis of selected TGF-β-responsive and EMT-associated proteins shows that markers induced by TGF-β are reduced by both CEL and ExoCEL, in H1299 (C) and A549 (D) with ExoCEL producing a more consistent suppression across targets. Data are normalized to beta-actin and expressed as average ± SD (n = 3). Statistical differences were evaluated using one-way ANOVA followed by Bonferroni's multiple-comparison test (*p < 0.05, **p < 0.01, ***p < 0.001 vs. control).

Epithelial-mesenchymal transition (EMT) is a fundamental biological process during development and cancer progression, in which epithelial cells acquire mesenchymal, fibroblast-like characteristics, leading to reduced cell-cell adhesion and increased motility 39. To study the effect of CEL in an EMT scenario, we selected H1299 cells due to their metastatic origin and portrayal of strong EMT marker changes and migration/invasion phenotypes. To assess the effect of celastrol (CEL) on TGF-β-induced EMT markers, we evaluated the expression of key EMT markers in H1299 lung cancer cell lines by Western blot analysis. Stimulation with TGF-β led to a marked downregulation of epithelial marker E-cadherin, and upregulation of epithelial marker ZO-1 and mesenchymal markers - N-cadherin, and β-catenin, confirming successful induction of the EMT program. In addition, Claudin-1, a tight junction protein essential for epithelial barrier function, was also downregulated upon TGF-β treatment (Figure 5A). These changes reflect key features of EMT, including the “cadherin switch”, characterized by loss of E-cadherin and gain of N-cadherin expression, which is a hallmark of invasive and metastatic tumor phenotypes 40.

Treatment with CEL, either alone or in combination with TGF-β, reversed many of these molecular alterations in a dose-dependent manner. CEL restored the epithelial markers E-cadherin, and Claudin-1 expression, suggesting a re-establishment of epithelial phenotype and junctional integrity. Concurrently, CEL suppressed the expression of N-cadherin, Vimentin, β-catenin, and ZO1, even in the presence of TGF-β (Figure 5A). A similar effect was observed in A549 cells (Figure S4). These findings indicate that CEL effectively inhibits both canonical and non-canonical EMT pathways, counteracting TGF-β-driven EMT in lung cancer cells.

To further confirm at the transcriptional level, we did qRT-PCR analysis of EMT-related genes in H1299 cells. Consistent with the Western blot results, TGF-β stimulation markedly reduced mRNA levels of E-cadherin and Claudin-1, while significantly upregulating N-cadherin, Vimentin, β-catenin, and ZO-1 (Figure 5A1-A6). Treatment with CEL reversed these changes in a dose-dependent manner, restoring epithelial markers (E-cadherin and Claudin-1) and suppressing mesenchymal markers (N-cadherin, Vimentin, β-catenin, and ZO-1), even in the presence of TGF-β. These results confirm that CEL not only modulates EMT marker proteins but also exerts consistent regulatory effects at the mRNA level, thereby reinforcing its ability to counteract TGF-β-driven EMT in lung cancer cells.

### CEL inhibits oncogenic signaling pathways in A549 cells

We examined the effects of CEL on critical oncogenic and apoptotic pathways in the presence and absence of TGF-β, a known promoter of EMT and tumor progression (Figure 5B). Cells were treated with TGF-β alone or co-treated with increasing concentrations of CEL. TGF-β exposure appeared to enhance KRAS protein expression, consistent with its known role in promoting oncogenic pathways. CEL treatment attenuated KRAS levels in a dose-dependent manner in A549 cells (Figure S5). Other downstream effectors, such as p-AKT, which are commonly activated by KRAS signaling, were also reduced following CEL treatment, further supporting its inhibitory effects on proliferative signaling. Total AKT levels remained stable, and β-actin confirmed equal protein loading.

Further analysis revealed that PI3K and c-Myc, two downstream effectors involved in cell proliferation and survival, were also suppressed following CEL treatment. TGF-β alone modestly upregulated PI3K and c-Myc expression, consistent with its role in promoting oncogenic phenotypes. However, co-treatment with CEL markedly reduced both PI3K and c-Myc levels in a concentration-dependent manner. Additionally, TGF-β stimulation elevated the expression of SMAD4, a central mediator of canonical TGF-β signaling. CEL treatment led to a dose-dependent reduction in SMAD4 protein levels, suggesting that CEL interferes with TGF-β-driven transcriptional responses. Collectively, these findings indicate that CEL disrupts both TGF-β/SMAD and KRAS-mediated signaling, potentially limiting tumor progression and EMT in A549 cells.

To complement the protein-level data, we assessed the transcript expression of KRAS, PI3K, c-MYC, and SMAD4 by qRT-PCR. Consistent with Western blot results, TGF-β treatment upregulated all four genes, reflecting activation of oncogenic and EMT-promoting pathways. CEL co-treatment produced a dose-dependent suppression of these transcripts, significantly reducing KRAS, c-MYC and PI3K mRNA levels and markedly downregulating SMAD4 compared with TGF-β alone (Figure 5B1-B4). These findings confirm that CEL not only attenuates oncogenic signaling at the protein level but also exerts parallel transcriptional repression, reinforcing its role in disrupting TGF-β-driven tumor progression.

### CEL- and ExoCEL-mediated suppression of TGF-β signaling

Consistent with the transcriptomic findings, Western blot analysis of selected EMT- and TGF-β-responsive proteins showed that markers upregulated following TGF-β treatment were markedly reduced upon treatment with CEL and its exosomal formulation (Figure 5C-D). While both treatments attenuated TGF-β-induced protein expression, ExoCEL generally produced a stronger and more consistent reduction compared with free CEL.

Notably, both CEL and ExoCEL effectively suppressed the expression of TGF-β-induced c-Myc, a key oncogenic regulator of cell proliferation and survival. The enhanced downregulation observed with ExoCEL suggests improved intracellular delivery and sustained bioavailability of CEL when administered via exosomes. Importantly, these data indicate that exosomal encapsulation preserves the molecular mechanism of CEL action while enhancing its functional efficacy at the protein level.

### Tumor-targeting of FA-Exo in lung-tumor bearing mice

**Figure 6:**
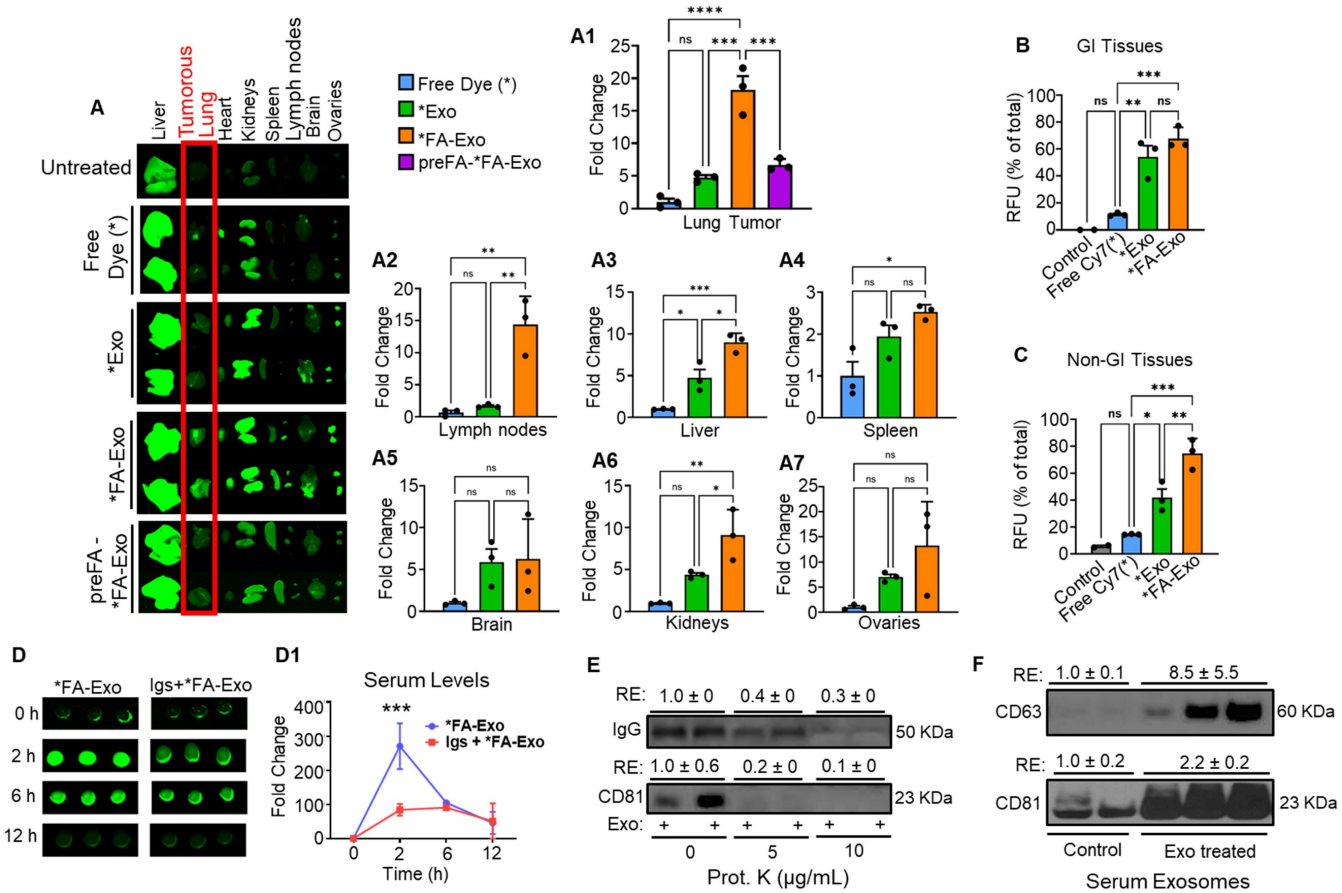
**Tumor targeting of FA-Exo in orthotopic lung tumor model.** Lung tumor-bearing female C57BL/6 mice (n=3) were treated with a single oral dose of free Cy7(*), *Exo and *FA-Exo (100,000 fluorescence units/animal). An untreated mouse served as a control for background fluorescence. An additional group was included in which mice were pretreated with two doses of free FA prior to administration of *FA-Exo. Mice were euthanized 12 h post-administration, and all major organs, including tumor-bearing lung tissue, were imaged *ex vivo* using an Odyssey LiCor CL system **(A)**. Fluorescence signals from each tissue were quantified, normalized to free dye, and expressed as fold change in bar diagrams (A1-A7). Panels B and C show relative fluorescence units (RFU) expressed as the percentage of total signal detected in GI **(B)** and non-GI tissues **(C)**. **FcRn-mediated oral uptake of FA-Exo.** FA-Exo (50,000 fluorescence units/dose) was orally administered to female C57BL/6 mice with or without IgG pretreatment (100 mg/kg, 1 h prior). To evaluate systemic uptake, blood was collected at various time points, and fluorescence intensity was measured from 5 µL of serum **(D)**. Fold change in fluorescence intensity was calculated and plotted (D1). **(E)** Immunoblot analysis showing reduced IgG and CD81 signals following Proteinase K treatment of exosomes. **(F)** Western blot detection of bovine exosomal markers CD81 and CD63 in serum-derived vesicles following oral administration, indicating recovery of intact exosomes in circulation. Data are presented as mean ± SD (n = 3 mice/group). One-way ANOVA followed by Bonferroni's multiple comparison test was used for statistical analysis (*p < 0.05, **p < 0.01, ***p < 0.001, ns = non-significant).

To evaluate tumor-targeting efficiency, C57BL/6 mice (n = 3) bearing orthotopic LLC-LUC2 lung tumors were orally administered free dye (cyanine 7 or Cy7), Exo-Cy7 (*Exo), or FA-Exo-Cy7 (*FA-Exo). After 12 h, *ex vivo* imaging using the Odyssey LI-COR CLx system revealed distinct biodistribution patterns across major organs (Figure 6A). At the tumor site, FA-Exo-Cy7 produced markedly stronger fluorescence than either free dye or Exo-Cy7, with a >4-fold increase compared to *Exo (p < 0.001) and an ~18-fold increase compared to free dye (p < 0.001) (Figure 6A1). The bar graphs further quantify these differences, showing consistent enhancement across all replicates (Fig 6A1-A7). In addition to the tumor, FA-Exo-Cy7 also accumulated at significantly higher levels in lymph nodes (14-fold, p < 0.01). However, the retention in liver and kidneys was approximately 2-fold (p < 0.05) relative to *Exo (Figure 6A2-A7).

To further validate folate receptor-mediated tumor targeting, mice were pretreated with excess free FA prior to FA-Exo-Cy7 dosing. FA pre-treatment resulted in a marked reduction in tumor-targeting compared to non-pretreated FA-Exo-Cy7 (Figure 6A, 6A1). Quantitative analysis confirmed a significant decrease in tumor accumulation following FA pretreatment (Figure 6A1). These findings provide direct evidence that the enhanced tumor accumulation of FA-Exo is driven by folate receptor-mediated uptake rather than nonspecific biodistribution.

When comparing signals across gastrointestinal (GI) and non-gastrointestinal (non-GI) tissues, FA-Exo exhibited levels comparable to Exo in the GI tract but showed markedly higher accumulation in non-GI tissues (Figure 6B & 6C). These findings indicate enhanced uptake or retention of FA-functionalized formulations in non-GI organs and collectively demonstrate that FA functionalization substantially improves the systemic retention and tumor-targeting capability of exosomes, likely through folate receptor-mediated uptake.

### FcRn-mediated uptake of FA-Exo upon oral administration

To determine the mechanism of GI absorption, we examined FA-Exo uptake in mice with or without FcRn receptor saturation using bovine Igs. In control mice, oral administration of FA-Exo resulted in a rapid increase in serum fluorescence, with ~270-fold elevation peaking at 2 h before gradually declining, indicating efficient entry of FA-Exo into systemic circulation. In contrast, FcRn-saturation in mice displayed markedly attenuated absorption, showing only ~85-fold and ~91-fold increases at 2 and 6 h, respectively (Figure 6D & 6D1). Considering the presence of Igs on the surface of exosomes, and high GI uptake observed in the biodistribution study, these findings strongly support FcRn involvement in FA-Exo oral uptake, while alternative absorption pathways cannot be excluded.

To further support the involvement of FcRn in FA-Exo absorption, we validated the presence of the IgGs on the exosomal surface. Proteinase K treatment of exosomes resulted in a marked (>70%) reduction of IgG signal, along with the expected decrease in the surface exosomal marker CD81, as shown by immunoblotting (Figure 6E). These findings confirm that IgG is predominantly on exosomes surface, making it accessible for interaction with FcRn.

### Exosomes remain intact in serum following oral administration

To determine if orally given exosomes could be detected in the circulation in an intact form, exosomes were isolated from serum of either vehicle-treated or exosome-treated mice. Serum-derived exosomes were analyzed by Western blot revealed the presence of bovine-specific exosomal markers CD81 and CD63 in serum samples collected from exosome-treated mice, whereas these markers were either not detected or had some non-specific signals in vehicle-treated controls (Figure 6F). These findings indicate that orally administered exosomes can cross the GI barrier and be recovered from the circulation while retaining exosomal protein markers.

### Tumor-targeting enhances anti-tumor activity

To determine whether FA-ExoCEL enhances therapeutic efficacy in the tumor microenvironment, an orthotopic lung tumor model was established using female NOD-SCID mice. Mice (n = 7-9 per group) were intrathoracically injected with 2 × 10⁶ Bioware® Brite A549 Red-Fluc lung cancer cells in Matrigel 22, 35, 36. After 12 d, once tumors were established, animals were randomized and treated orally three times per week with FA-ExoCEL (8 mg/kg) or vehicle (PBS). Other treatment arms, including Exo alone, free CEL, and ExoCEL, were previously evaluated in subcutaneous (*s.c.*) tumor models and were therefore not included in this study 23. Tumor progression was monitored weekly using *in vivo* bioluminescent imaging.

**Figure 7:**
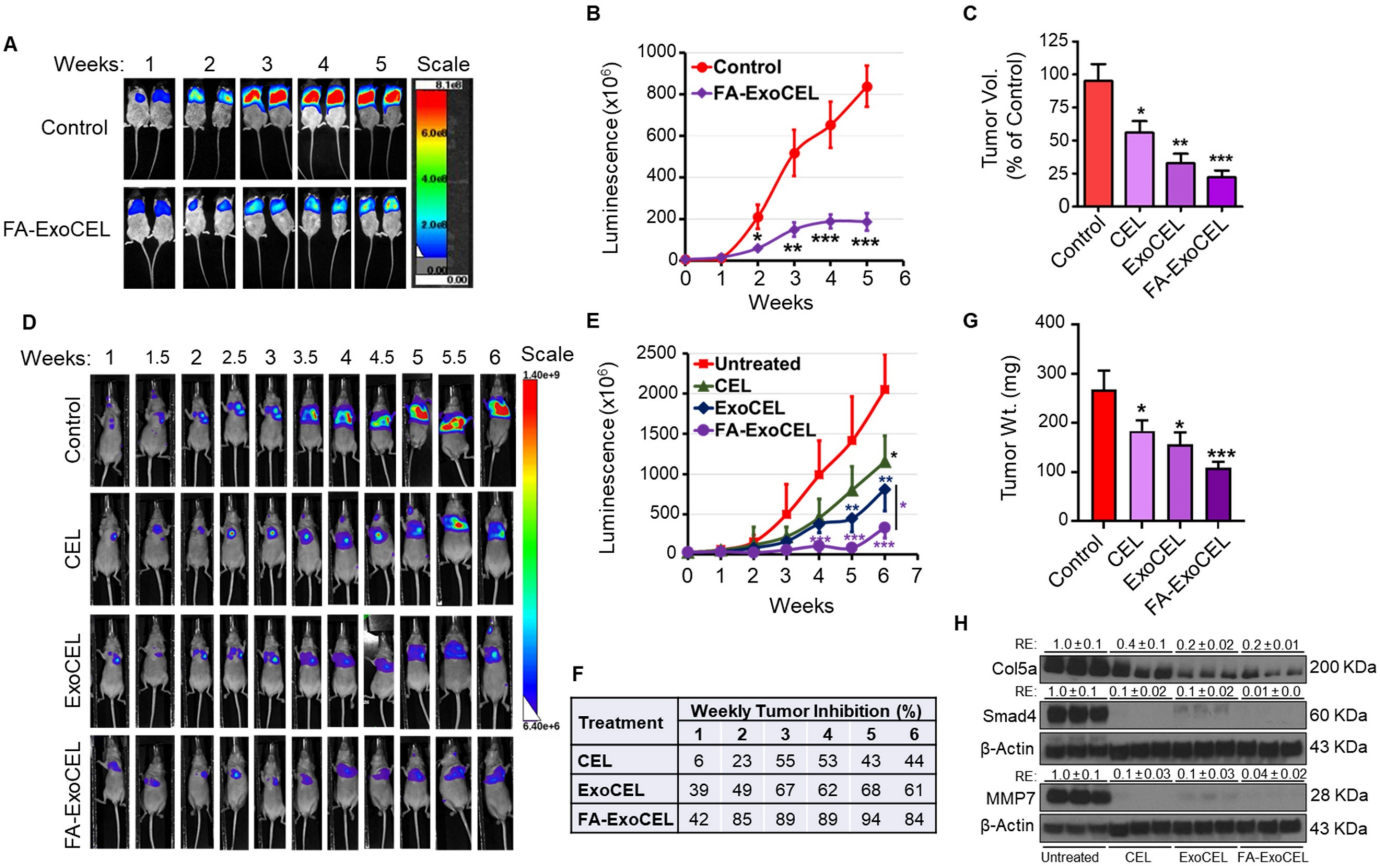
** Oral administration of FA-ExoCEL significantly inhibits orthotopic A549 lung tumor growth *in vivo*. (A)** Representative bioluminescent images of NOD-SCID mice bearing orthotopic A549-Red-FLuc tumors. Mice were treated with either PBS (control) or FA-ExoCEL (8 mg/kg) via oral gavage three times per week for 5 weeks. Tumor progression was monitored weekly using *in vivo* imaging. **(B)** Quantitative analysis of bioluminescent signal over 5 wk demonstrates significant tumor growth inhibition in FA-ExoCEL treated animals compared to control (p < 0.001 at weeks 3-5). **(C)** Tumor growth inhibition by CEL, ExoCEL, and FA-targeted ExoCEL was compared using data from two independent studies. In the previous study 23, A549 subcutaneous tumors were treated with CEL and ExoCEL, while the current study evaluated FA-ExoCEL in an orthotopic lung tumor model. Tumor growth inhibition was calculated relative to the respective control groups, which were set at 100%. **(D)** Representative bioluminescent images from an orthotopic lung tumor study comparing oral administration of free CEL, ExoCEL, and FA-ExoCEL within the same experiment. **(E)** Quantitative analysis of tumor-associated bioluminescent signals over time demonstrating progressively greater tumor suppression with ExoCEL compared with CEL, and the most pronounced inhibition with FA-ExoCEL. **(F)** Weekly tumor growth inhibition (%) calculated for each treatment group, highlighting antitumor efficacy of FA-ExoCEL relative to CEL and ExoCEL. **(G)** Western blot analysis of tumor tissues showing reduced expression of selected oncogenic and EMT-associated proteins in ExoCEL- and FA-ExoCEL-treated groups compared with free CEL, consistent with enhanced intratumoral drug activity. Statistical significance was evaluated using two-way ANOVA followed by Bonferroni post hoc test. Values are shown as mean ± SEM (n = 7-9 mice per group). *p < 0.05, **p < 0.01, ***p < 0.001 vs. control.

As shown in Figure 7A, FA-ExoCEL treatment resulted in a marked reduction in luminescent signal intensity compared to controls, indicating reduced tumor burden. Quantitative analysis revealed progressive and significant tumor growth inhibition with FA-ExoCEL over the five-week period, reaching 77% inhibition by week 5 (Figure 7B). The weekly inhibition data demonstrate sustained efficacy, with tumor suppression evident from week 2 onward.

In a comparative analysis across two independent studies (Figure 7C), the efficacy of FA-ExoCEL in the orthotopic model surpassed that of both CEL and ExoCEL treatments in the *s.c.* tumor model using the same A549 cell line. These results support the added benefit of FA-Exo mediated targeting in enhancing ExoCEL therapeutic activity. Statistical analysis using two-way ANOVA followed by Bonferroni post hoc test confirmed significant tumor suppression with FA-ExoCEL (*p<0.05, **p < 0.01, ***p < 0.001).

To directly compare the therapeutic efficacy of free CEL, ExoCEL, and FA-ExoCEL within the same experimental setting, an additional orthotopic lung tumor study was performed using female athymic nude mice. As shown in Figure 7D, live imaging showed treatment-dependent differences in tumor progression. Control animals exhibited a rapid increase in luminescent signal over time, whereas CEL treatment resulted in only modest tumor growth suppression. In contrast, ExoCEL treatment demonstrated a more pronounced reduction in tumor burden, while FA-ExoCEL consistently showed the strongest suppression of tumor growth across all time points. Quantitative analysis of luminescence signals confirmed significantly greater tumor inhibition with ExoCEL compared with free CEL, and further enhancement with FA-ExoCEL (Figure 7E & F). FA-ExoCEL exhibited the highest and most durable inhibition (Figure 7F), reaching >80% tumor growth inhibition and significant reduction in tumor weight (Figure 7G). Importantly, all treatment regimens were well tolerated, with no significant changes in body weight or observable signs of systemic toxicity.

To validate the transcriptional changes observed following CEL-based treatments, we examined the protein expression of selected markers in tumor tissues harvested from the *in vivo* efficacy study by western blot analysis. Consistent with the antitumor responses observed, both CEL and ExoCEL treatment resulted in reduced expression of extracellular matrix- and invasion-associated proteins, including COL5A1 and MMP7 (Figure 7H). In agreement with our *in vitro* findings, Smad4 expression was also downregulated in tumor tissues, the decrease was more substantial in cells treated with ExoCEL, suggesting enhanced inhibition of TGF-β-related signaling at the protein level.

### Systemic toxicity evaluation of FA-ExoCEL

**Figure 8:**
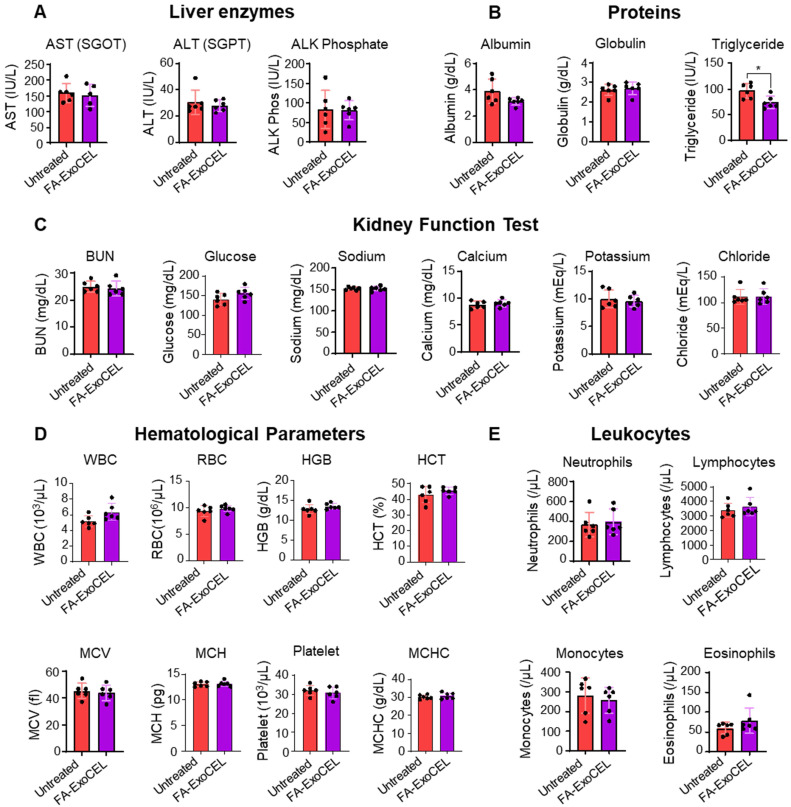
**Toxicity assessment of FA-ExoCEL**. Wild-type female C57BL/6 mice (n = 6 per group) were orally treated with vehicle or FA-ExoCEL (8 mg/kg b. wt., 3 times/week) for four weeks. Serum biochemical markers of liver function **(A)**, proteins levels **(B)** kidney function **(C)**, hematopoietic indices **(D)**, and metabolic parameters **(E)** were analyzed at the end of treatment. No significant differences were observed between groups for liver, kidney, or hematopoietic markers. A modest reduction in serum triglycerides was detected in FA-ExoCEL treated mice, but values remained within physiological limits. Data are presented as mean ± SD. Statistical analysis was performed using Student's t-test (*p < 0.05).

To assess systemic safety, WT female C57BL/6 mice were orally administered FA-ExoCEL at 8 mg/kg body weight, three times per week for four weeks; vehicle-treated mice served as controls. No gross signs of toxicity were observed, and animals maintained normal activity, diet intake, and body weight gain. Biochemical analysis showed no significant differences in liver function (ALT, AST) or kidney function (BUN, creatinine) between groups. Hematopoietic indices, including WBC, RBC, HGB, HCT, and PLT, remained within normal physiological ranges. Among metabolic parameters, only serum triglycerides were modestly reduced in the FA-ExoCEL group, but values remained within the physiological range. All other biochemical markers, including cholesterol, glucose, and albumin, were comparable between groups (Figure 8A-E). Collectively, these findings indicate that repeated oral administration of FA-ExoCEL is well tolerated and does not induce systemic toxicity under the tested conditions.

### CEL enhances *in vitro* efficacy of chemo drug

**Figure 9:**
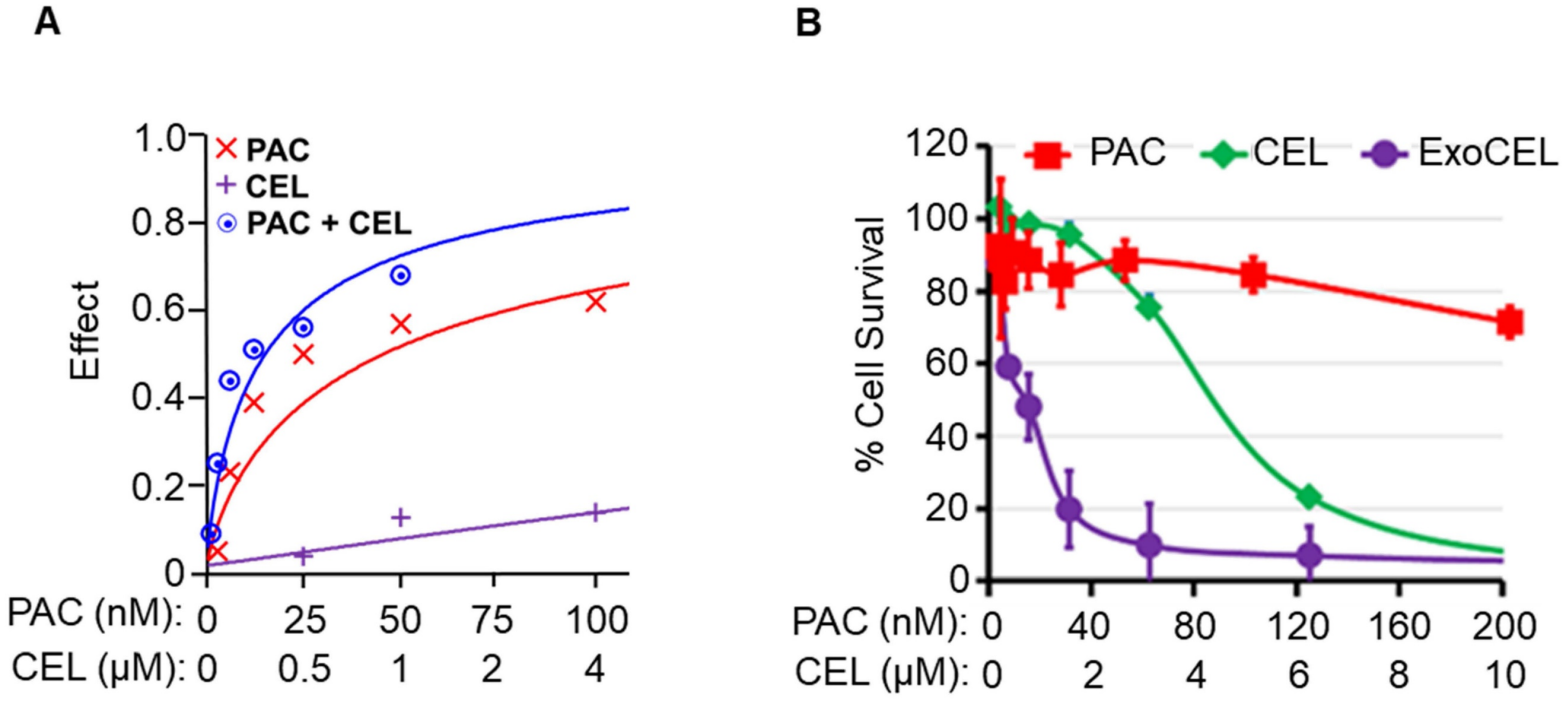
**CEL enhances paclitaxel efficacy and overcomes drug resistance in A549TR cells. (A)** Drug interaction analysis in A549TR cells treated with paclitaxel (PAC), CEL or their combination. The combination treatment showed greater inhibitory effect than either drug alone, indicating synergism. Drug effect was modeled using median-effect analysis calculated using CalcuSyn software. **(B)** Dose-dependent cytotoxicity in A549TR cells treated with PAC alone, free CEL, or ExoCEL. ExoCEL showed significantly enhanced cytotoxicity compared to both free CEL and PAC alone, reducing cell viability even in the resistant cell line. The Exo alone showed only less than 10% growth inhibition and not included in for clarity. Data are presented as mean ± SD from triplicate samples.

To determine if CEL enhances activity of standard chemo drug PAC, we treated A549 lung cancer cells with CEL and PAC, alone and in combination and measured cell survival by MTT assay after 72 h. Our data indicate strong dose-dependent synergism between CEL and PAC against A549 NSCLC cells. In these studies, our analysis of synergism was achieved with the median-effect equations and combination index (CI) methods developed by Chou et al 41. Data (Figure 9A) showed that the combination effect was greater than individual compounds in lung cancer cell lines. The combination index (CI-plot) was less than 1 indicating synergism as analyzed by Calcusyn software.

### Exo formulations enhance activity of CEL in drug-resistant lung cancer cells

ExoCEL showed over 2-3-fold reduction in the IC**_50_** values compared with free CEL 23. MTT assay showed higher growth inhibition by ExoCEL vs. CEL against drug-resistant (IC50 0.63 µM vs 5 µM; p<0.001) cells (Figure 9B). The enhanced activity of the ExoCEL could result from greater cell uptake and/or slow release or retention of CEL 23. The anti-proliferative activity of CEL corroborates the inhibition of A549- and A549TR-mediated colony formation; the effect of ExoCEL was greater than free CEL and inhibited the colony formation completely at 1 µM concentrations (not shown).

## Discussion

Our optimized differential centrifugation protocols 24 consistently yielded high quantities of exosomes with desirable physicochemical characteristics, including nanoscale size (30-150 nm), low PDI, and negative zeta potential, all indicative of a stable colloidal system. The isolated exosomes expressed canonical exosomal markers, including CD81 and Alix, while non-exosomal markers such as calnexin and GM130 were not detected. This profile confirms the exosomal identity and purity of the preparations and minimizes the likelihood that the observed biological effects arise from contaminating cellular debris or organelle fragments. Importantly, exosome preparations from powdered colostrum were comparable in size distribution and surface marker expression (CD63, CD81, Alix, Tsg101), highlighting the reproducibility and flexibility of our isolation approach 24, 37.

A key innovation in our study is the successful loading of CEL, a hydrophobic compound with known therapeutic potential, into colostrum exosomes. The modest increase in particle size and PDI post-loading suggests that the exosomal membrane tolerates drug incorporation without compromising stability, an observation consistent with other exosomal drug delivery studies 42. To elucidate the mechanism of drug loading, we investigated intrinsic protein fluorescence changes upon CEL and FA incorporation. The marked quenching (~45% and 80%) of tryptophan-associated fluorescence supports our hypothesis that hydrophobic interactions between the exosomal membrane proteins and drug molecules are a primary mode of loading. This aligns with previous reports demonstrating fluorescence quenching as an indicator of drug-protein interactions, such as in the case of albumin-drug binding studies 43. While hydrophobic interactions with surface proteins appear to be a dominant mechanism, we acknowledge the possibility of drug encapsulation within the lipid bilayer or exosomal lumen, warranting further studies using fluorescence resonance energy transfer (FRET), cryo-EM, or mass spectrometry-based mapping techniques.

Proteinase K digestion studies indicate that a substantial fraction of CEL is bound to exosomal surface proteins via hydrophobic interactions, while incomplete removal after protease treatment suggests that additional CEL is embedded within the lipid bilayer or protected inside the lumen. This dual loading is beneficial for oral delivery, as surface-associated drug may facilitate rapid release and cellular interaction, while membrane- or lumen-associated CEL likely contributes to sustained stability and controlled release under physiological conditions.

CEL is highly lipophilic and poorly water soluble and therefore, its release from FA-ExoCEL is dependent on the solubilization capacity of the release medium and not the vesicle instability alone. The highest release of CEL observed in FeSSIF shows its strong sink conditions due to bile salt-lecithin mixed micelles, while FaSSIF supports moderate release because of lower micellar content. In contrast, FaSSGF provides minimal solubilization capacity for CEL, resulting in slow release, whereas the intermediate release in PBS likely reflects gradual dissociation of CEL from exosomal surfaces. Overall, this release behavior confirms the stability of ExoCEL under gastric conditions and higher release in the intestinal environment.

CEL has gathered significant attention for its potent anti-inflammatory and anti-cancer properties. In this study, we demonstrate that CEL effectively inhibits the proliferation of NSCLC cells *in vitro* and suppresses tumorigenic phenotypes through multiple mechanisms, including modulation of oncogenic signaling, and epithelial-mesenchymal transition (EMT). Our findings show that CEL exerts dose- and time-dependent anti-proliferative effects in H1299 (IC₅₀ 0.63 µM) and A549 cells (IC₅₀ 1.8 µM) 23. The anti-proliferative effects of CEL and ExoCEL were further validated through colonogenic assays in both drug-sensitive and resistant lung cancer cells.

While both formulations significantly suppressed colony formation, ExoCEL demonstrated improved efficacy in H1299 cells, suggesting enhanced uptake or drug retention in certain cellular contexts. However, in A549 and A549-TR cells, CEL and ExoCEL exhibited comparable activity, indicating that exosomal encapsulation may not universally enhance delivery but could confer cell type-specific advantages.

Exosomal formulation demonstrated improved intracellular delivery of the payload, as evidenced by higher uptake of Exo-Cy7 in both A549 and H1299 cells. The intracellular uptake and trafficking of milk-derived exosomes have been extensively characterized in our earlier studies, demonstrating energy-dependent endocytosis and delivery of functional cargo to recipient cells 28, which can bypass limitations associated with free small-molecule diffusion. The uptake was higher in H1299 cells compared to A549 cells, which could be due to leaky nature of H1299 cells and could be the reason of higher sensitivity to ExoCEL. Importantly, increased uptake provides a mechanistic basis for the improved biological activity observed with ExoCEL and FA-ExoCEL in downstream functional assays, and *in vivo* tumor inhibition. The selective modulation of claudin genes upon CEL treatment suggests that CEL disrupts tight junction integrity in a targeted manner. The downregulation of both *CDH1* and *CDH2* indicates that CEL destabilizes epithelial adhesion as well as mesenchymal signaling, potentially disrupting hybrid EMT states and limiting metastatic plasticity. Non-clustered protocadherins (PCDHs) are often epigenetically silenced in cancers and may act as tumor suppressors 44. In this context, although there is no established literature on *PCDH19*, we observed that among all protocadherins, *PCDH19* was significantly upregulated following CEL treatment. Further investigation is warranted to elucidate the implication of *PCDH19* upregulation in lung cancer.

MMPs are strongly associated with poor prognosis in lung cancer due to their role in promoting tumor invasion through enhanced extracellular matrix degradation 45. Thus, the CEL-induced upregulation of *MMP1* and *MMP3* merits further investigation. Conversely, the downregulation of *MMP7*, a known oncogenic factor and *MMP24* correlated with the inhibition of cancer cell proliferation and invasion observed in our *in vitro* studies 46. Suppression of *MMP24* may further enhance therapeutic efficacy by limiting tumor invasion and metastasis, thereby improving patient outcome 47.

These gene expression changes are consistent with the downregulated biological processes identified in our enrichment analysis. Disruption of tight junctions has been associated with reduced lung cancer proliferation and invasion 48. The modulation of EMT-related genes, along with downregulation of genes involved in tight junction and cell-cell junction assembly and organization, may impair tumor cell adhesion and polarity. This could reduce metastatic potential and enhance drug penetration into tumor tissue 49, 50. Furthermore, the observed downregulation of processes such as spindle assembly and meiotic nuclear division may impair mitotic fidelity, leading to increased cancer cell death 51. Enrichment analysis also revealed vasoconstriction as a downregulated process, which may enhance tumor perfusion, improve drug delivery, and facilitate immune cell infiltration 52.

We observed that CEL treatment downregulated the gene expression of *AKT2, AKT3* and *SMAD6 in vitro*. Reduced expression of *AKT2* and *AKT3* in lung cancer has been associated with decreased tumor cell proliferation, motility, invasion, and angiogenesis 53. Similarly, downregulation of SMAD6, an inhibitory SMAD, may restore canonical TGF-β/SMAD signaling, thereby exerting tumor-suppressive effects such as cell cycle arrest and apoptosis in lung cancer cells, counteracting tumor progression and potentially improve therapeutic outcomes 54. Collectively, these changes suggest that CEL suppresses oncogenic signaling while enhancing tumor-suppressive activity.

At the protein level, however, we found that CEL treatment resulted in downregulation of Smad4, with minimal change in expression under co-treatment conditions. Although loss of Smad4 has been linked with metastasis and poor prognosis, TGF-β/Smad4 signaling is also recognized as protective in early stages of tumorigenesis 55 These apparently divergent effects highlight the complexity of TGF-β/SMAD signaling in lung cancer. Further studies are warranted to elucidate how CEL modulates these pathways at both the transcriptional and protein levels, and to clarify the stage-specific role of Smad4 in mediating its antitumor effects.

Collectively, the significant upregulation of *SENS2, DEDD2, BTG2, STX3* and *GADD45G* reflect on cancer cell's response to drug-induced stress and may contribute to tumor suppression 56-59. CEL treatment also led to downregulation the collagen-related genes (*COL5A1, COL7A1, and COL1A1*), which are key elements of the extracellular matrix with essential biological functions in tumor progression 60. Although their specific roles in lung cancer are not fully characterized, they hold potential as prognostic markers. Additionally, downregulation of *FGB* and *SULT2B1* suggests disruption of metabolic pathways critical for tumor survival, including tumor-associated thrombosis, angiogenesis, and lipid metabolism 61, 62.

Importantly, CEL displayed minimal cytotoxicity toward non-tumorigenic bronchial epithelial (Beas2B) and breast epithelial (MCF10A) cells, with a >5-fold selectivity index, consistent with earlier reports of CEL's preferential cytotoxicity toward cancer cells over normal tissue. This selective cytotoxicity strengthens CEL's candidacy as a targeted therapeutic agent with a potentially favorable safety profile. Both RNA-seq and protein analyses showed that CEL and ExoCEL reverse TGF-β-induced EMT in lung cancer cells. While CEL and ExoCEL elicited largely overlapping transcriptional responses, ExoCEL produced enhanced or more consistent protein suppression, suggesting it to be a consequence of improved intracellular uptake rather than a change in mechanism of action.

In addition to its cytostatic effects, CEL and ExoCEL effectively suppressed lung cancer cell migration, as demonstrated in the wound healing assay. Notably, both CEL and its exosomal formulation inhibited TGF-β-induced EMT, a key process implicated in cancer metastasis and drug resistance. CEL reversed TGF-β-mediated upregulation of mesenchymal markers (N-cadherin, Vimentin, β-catenin) while restoring epithelial markers (E-cadherin and Claudin-1), effectively reversing the “cadherin switch,” a hallmark of EMT 63. These findings align with studies in other cancer models, where CEL inhibits EMT by modulating TGF-β/Smad and MAPK signaling 64.

We 23 and others have shown CEL to induce ER stress and inhibit Hsp90 65-67. The major apoptotic regulator, p53, is increased in response to ER stress 68-70 and inhibition of Hsp90 to promote p53-dependent apoptosis 71, 72. CEL inhibits TGF-β1-induced EMT by inhibiting Snail and regulating E-cadherin expression 6, 73 in A549 cells. In agreement to this report, our preliminary findings indicated CEL's ability to inhibit TGF-β-induced migration of lung cancer cells via suppression of EMT markers. In a recent study CEL shows direct inhibition of cMyc-Max heterodimerization 74.

KRAS mutations are among the most common drivers in NSCLC and are associated with poor prognosis and resistance to therapy 75. At the molecular level, CEL reduced KRAS protein expression in A549 cells in a dose-dependent manner. Concomitantly, CEL treatment restored apoptotic signaling as indicated by increased cleavage of PARP and caspase-3. Overall, these results suggest that CEL inhibits tumor growth through a multifaceted mechanism involving suppression of oncogenic KRAS signaling, inhibition of EMT, and induction of apoptosis. The exosomal formulation retained or enhanced these effects in selected cell lines, supporting the use of milk-derived exosomes as a biocompatible delivery system for hydrophobic drugs like CEL.

The therapeutic potential of CEL is further strengthened by strategies that enhance its tumor specificity and overcome drug resistance. FA-targeting exploits the overexpression of folate receptors on various tumors, including NSCLC, to selectively direct therapeutic agents to cancer cells 76. Exosomes are particularly amenable to functionalization due to their lipid bilayer and surface proteins, making them an ideal vehicle for receptor-mediated drug delivery. Moreover, FA-functionalization caused only minor, statistically insignificant changes in particle size and did not alter morphology or polydispersity.

Our biodistribution data clearly demonstrate that FA-Exo achieves superior tumor targeting compared with both free dye and non-targeted exosomes, with more than a four-fold increase in tumor accumulation relative to Exo and nearly an eighteenfold increase over free dye. This striking improvement highlights the role of folate receptor functionalization in enhancing systemic delivery and tumor localization, consistent with prior reports of FRα overexpression in NSCLC 32, 77. The additional enrichment of FA-Exo in lymph nodes may further point to an opportunity for modulating tumor-immune cross talk, as lymphoid tissues are central to anti-tumor immune responses. Higher tumor accumulation of FA-Exo was attenuated by FA pretreatment, confirming receptor-mediated targeting. The ability to achieve selective tumor enrichment while limiting off-target accumulation is particularly important for oral therapies.

Importantly, FA-Exo exhibited uptake levels in GI tissues comparable to non-targeted exosomes but showed significantly greater accumulation in non-GI organs, indicating efficient translocation across the gut barrier and systemic stability. Exosomes are naturally equipped with a lipid bilayer that confers resistance to enzymatic degradation in the GI tract, enabling them to maintain integrity after oral administration. In addition, the enrichment of CD47 on the surface of bovine colostrum-derived exosomes may provide a “don't eat me” signal that helps them evade rapid clearance by macrophages, thereby extending their circulation time and enhancing tumor targeting 12, 13. However, because the functional compatibility of bovine CD47 with murine SIRPα was not directly evaluated, this mechanism should be considered potentially contributory rather than definitive, and future studies will be required to clarify the extent of cross-species CD47-SIRPα signaling. Together, these features likely explain the observed biodistribution patterns and underscore the translational promise of FA-Exo as a safe and effective oral nanotherapeutic platform for lung cancer treatment.

Another important finding from our study is the demonstration that GI absorption of FA-Exo is at least partly mediated by the neonatal Fc receptor (FcRn). FcRn is highly expressed in the intestinal epithelium and facilitates transcytosis of immunoglobulins and IgG-bound ligands across mucosal barriers 78, 79. Our experiments with Ig pretreatment, which competitively saturates FcRn, showed a marked reduction in systemic fluorescence following oral administration of FA-Exo, strongly implicating transport into the gut epithelium is mediated by interaction between endogenous Igs surface bound to exosomes and the Fc receptors present in the GI tract 80. This is consistent with earlier work showing that milk-derived exosomes exploit FcRn-mediated transport for efficient passage across the intestinal barrier 81. By engaging FcRn, FA-Exo gain a mechanism to escape luminal degradation, crosses the epithelial barrier, and enter systemic circulation, thereby contributing to their favorable biodistribution and tumor-targeting properties. Detection of bovine exosomal markers CD81 and CD63 in serum following oral administration confirms that intact exosomes can enter systemic circulation. Furthermore, FcRn saturation experiments using IgG significantly reduced FA-Exo uptake, while Proteinase K digestion confirmed that IgG is surface-exposed on exosomes. Together, these findings support a role for FcRn-mediated transcytosis in facilitating oral absorption, while we can rule out that additional uptake pathways may also contribute.

In addition to FcRn-mediated uptake, the physicochemical properties of bovine colostrum-derived exosomes provide distinct advantages for oral delivery. Their natural lipid bilayer protects encapsulated cargo from enzymatic degradation and the highly acidic gastric environment, allowing them to maintain structural integrity during GI transit. Indeed, several studies have shown that milk-derived exosomes survive low pH and enzymatic digestion, underscoring their suitability as oral nanocarriers 82.

In our study, functionalization of ExoCEL with FA markedly enhanced its antitumor efficacy in an orthotopic lung tumor model, leading to nearly 80% tumor growth inhibition in just five weeks. This represents a substantial improvement over previously reported effects of CEL or ExoCEL in subcutaneous models, indicating that folate-mediated targeting enhances drug accumulation in the tumor microenvironment. The increased efficacy of FA-ExoCEL observed in our study supports previous reports where folate-decorated nanoparticles enhanced the biodistribution and tumor-selective uptake of anti-cancer agents 22. Additionally, in our previous study, CEL did not show any sign of toxicity when C57BL/6 mice were treated with either vehicle or CEL (8 mg/kg bw) daily for 15 d. The Exo- and CEL-treated animals showed no changes in the liver/kidney functions and hematopoietic parameters 23, 24.

The orthotopic lung tumor study with side-by-side comparison provided direct evidence that exosomal delivery and folate targeting significantly improve the oral antitumor efficacy of CEL. While free CEL showed limited activity, ExoCEL and FA-ExoCEL consistently achieved the highest tumor inhibition. Importantly, this enhanced efficacy was observed without observed systemic toxicity. Molecular analysis of tumor tissues further confirmed stronger suppression of oncogenic and EMT-associated proteins in ExoCEL- and FA-ExoCEL-treated groups, aligning mechanistic findings with therapeutic efficacy.

Moreover, our data demonstrates that CEL synergizes with PAC, a frontline chemotherapeutic agent for lung cancer. The strong dose-dependent synergy observed indicates that CEL enhances PAC's cytotoxicity, potentially through complementary mechanisms such as suppression of NF-κB and Hsp90 activity (CEL targets) alongside PAC-induced microtubule stabilization. These findings are in line with prior studies showing that CEL can sensitize cancer cells to chemotherapeutic agents, including doxorubicin, cisplatin, and bortezomib 83, 84. The ability to use lower drug doses in combination regimens could reduce toxicity while maintaining efficacy, an important consideration for future clinical applications.

Notably, ExoCEL demonstrated superior activity in drug-resistant A549-TR cells, with IC₅₀ values reduced by nearly 8-fold compared to free CEL. Drug resistance remains a major limitation in NSCLC treatment, and the ability of ExoCEL to overcome this barrier may result from enhanced cellular uptake, endosomal escape, or sustained release properties of the exosomal formulation 84. The complete inhibition of colony formation by ExoCEL at low micromolar concentrations further supports its therapeutic promise, even in chemoresistant contexts.

These findings are further supported by the rationale for folate receptor-mediated targeting in drug-resistant NSCLC. FRα is known to be highly expressed in a substantial subset of NSCLC, including A549 cells, and folate receptor-targeted nanocarriers have shown enhanced uptake and efficacy in resistant tumor models 85. Although direct FRα modulation was not assessed in our A549-TR cells, it is reasonable to expect that FA-ExoCEL benefits from increased folate receptor-mediated uptake, contributing to its ability to overcome chemoresistance.

While bovine colostrum-derived exosomes have a favorable safety profile and are widely consumed as part of the human diet, their xenogeneic origin does introduce regulatory considerations that will need to be addressed before clinical translation. These include careful evaluation of immunogenicity, standardization of large-scale manufacturing, and clarity around regulatory classification, which are the focus of ongoing and future studies.

Collectively, these findings underscore the translational potential of ExoCEL, particularly in its folate-targeted form, as a multi-functional nanotherapeutic capable of overcoming drug resistance, enhancing tumor targeting, and synergizing with conventional chemotherapies. These strategies offer a compelling avenue to improve treatment outcomes for patients with aggressive and refractory lung cancers. Taken together, our findings position bovine colostrum-derived exosomes as an attractive, scalable, and biocompatible delivery platform for oral administration of CEL.

## Supplementary Material

Supplementary figures and tables.

## Data Availability

All data supporting the findings of this study are included within the article and its Supplementary Materials. The RNA-sequencing data generated in this study have been deposited in the GEO repository under accession number GSE318992. Additional original data are available from the corresponding author upon request.

## Abbreviations

NSCLC: Non-small cell lung cancer
PDI: Poly dispersity index
CEL: Celastrol
ExoCEL: CEL loaded exosomes
FA-ExoCEL: CEL loaded FA-functionalized exosomes
FA: Folic acid
EMT: Epithelial-to-mesenchymal transition
STAT3: Signal transducer and activator of transcription 3
JNK 1/2: Janus-activated kinase-1 and -2
EVs: Extracellular vesicles
SIRPα: Signal regulatory protein-α
FRα: Folate receptor alpha
FRβ: Folate receptor beta
TAMs: Tumor-associated macrophages
MWCO: Molecular weight cut off
PBS: Phosphate buffer saline
AFM: Atomic force microscopy
UPLC: Ultra-performance liquid chromatography
Cy7: Cyanine-7
ACN: Acetonitrile
PDA-UV: Photodiode array UV detector
IgGs: Immunoglobulin G
Igs: immunoglobulins
FaSSGF: Fasted state simulated gastric fluid
FaSSIF: Fasted state simulated intestinal fluid
FeSSIF: Fed state simulated intestinal fluid
Exo-Cy7: Cy7-labeled exosomes
QC: Quality control
FcRn: Neonatal Fc receptors
PCA: Principal component analysis
SENP2: Stress-activated endoplasmic reticulum-nuclease 2
DEDD2: Death effector domain containing 2
BTG2: B-cell translocation gene 2
STX3: Syntaxin 3
GADD45G: Growth arrest and DNA damage inducible gamma
FGB: Fibrinogen beta chain
COL5A1: Collagen type V alpha 1 chain
COL7A1: Collagen type VII alpha 1 chain
COL1A1: Collagen type I alpha 1 chain
SULT2B1: Sulfotransferase family 2B member 1
MMPs: Matrix metalloproteinases
GO: Gene ontology
GI: Gastrointestinal
non-GI: Non-gastrointestinal
CI: Combination index
FRET: Fluorescence resonance energy transfer
PCDHs: protocadherins
