# Supplementary material for: Tumor-targeted Exosomal Delivery of Celastrol for Enhanced Therapeutic Efficacy in NSCLC
Source: Theranostics. 2026 Mar 3;16(10):5125–49. doi: 10.7150/thno.125096 (PMC13080336; doi:10.7150/thno.125096)
Supplement: Supplementary file 1 — Supplementary figures and tables. [file thnov16p5125s1.pdf]

Supplementary Data

Tumor-targeted exosomal delivery of celastrol for enhanced therapeutic efficacy in NSCLC

Disha Nagesh Moholkar, Raghuram Kandimalla, Mohd Saeed, Yaseera Arif, Neha Tyagi, Richa Singhal, Al-Hassan Kyakulaga, Amir Saeed, Margaret Wallen, Ramesh Gupta and Farrukh Aqil*

Table S1. Characterization of Exo, ExoCEL and FA-ExoCEL

Formulation	Batch #	Size (nm)	PDI	Zeta Potential
Exosomes	1	62 ± 5.2	0.282	- 15.4
	2	69 ± 6.1	0.251	- 14.9
	3	66 ± 5.9	0.263	- 16.3
	**Average**	**66 ± 7.7**	**0.265 ± 0.02**	**-15.53 ± 0.71**
ExoCEL	1	88 ± 4.9	0.301	- 17.2
	2	84 ± 4.8	0.292	- 12.9
	3	77 ± 6.2	0.272	- 14.6
	**Average**	**83 ± 5**	**0.288 ± 0.01**	**-14.9 ± 2.16**
FA-ExoCEL	1	82 ± 7.1	0.298	- 15.1
	2	98 ± 6.4	0.267	- 15.9
	3	93 ± 5.9	0.269	- 19.3
	**Average**	**91 ± 8**	**0.278 ± 0.02**	**-16.7 ± 2.23**

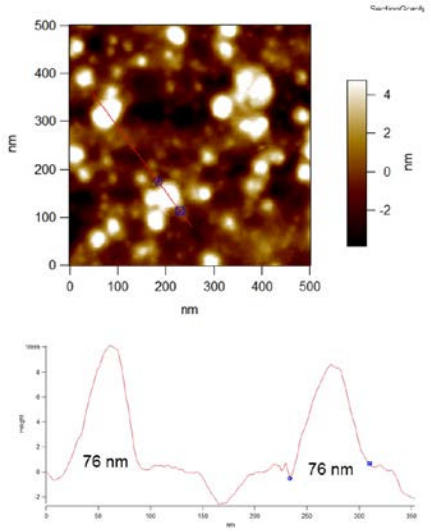

Figure S1. Atomic force microscopy (AFM) image showing morphology and nanoscale size distribution of exosomes and their measurement. This image is same as Figure 1C, except that it shows size measurement.

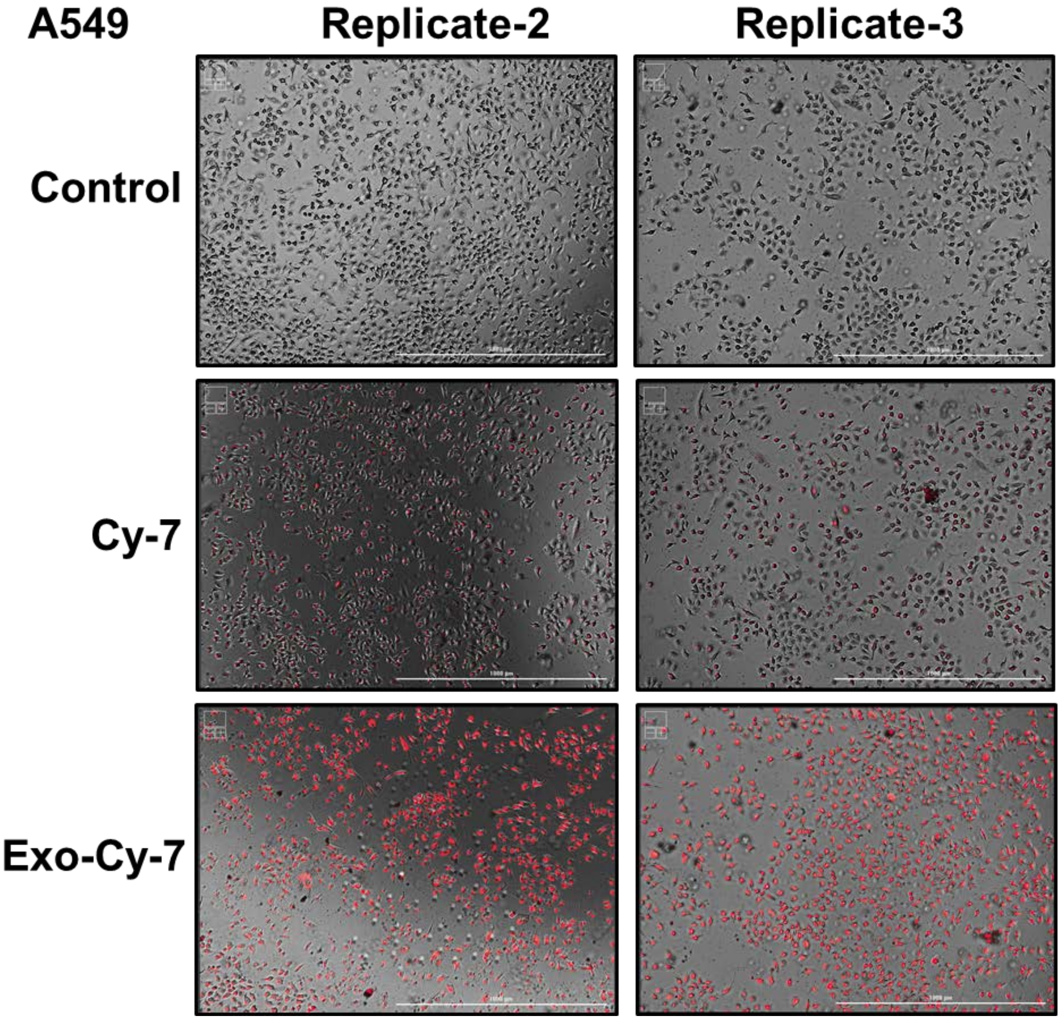

Figure S2: Cell uptake and internalization of payload in A549. Cells were treated for 4 h with either free Cy7 or ExoCy7 keeping the fluorescent intensity constant as 5000 RFU/well. Ultimately, the wells were imaged using the Lionheart-FX automated microscope (Agilent technologies, Santa Clara, CA). Representative images are shown in main Figure 2H and remaining two replicates are shown here.

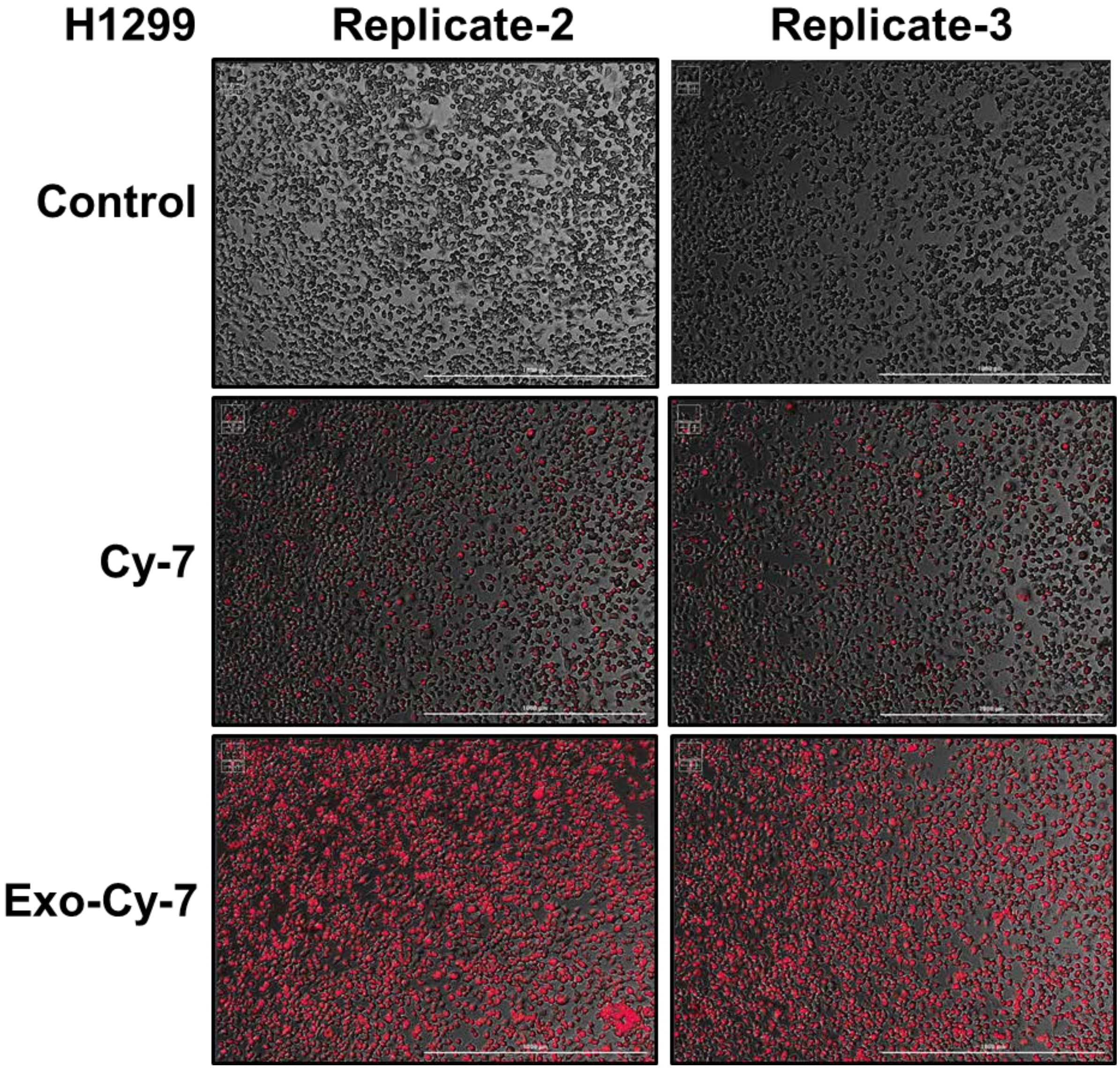

Figure S3: Cell uptake and internalization of payload in H1299. Cells were treated for 4 h with either free Cy7 or ExoCy7 keeping the fluorescent intensity constant as 5000 RFU/well. Ultimately, the wells were imaged using the Lionheart-FX automated microscope (Agilent technologies, Santa Clara, CA). Representative images are shown in main Figure 2H and remaining two replicates are shown here.

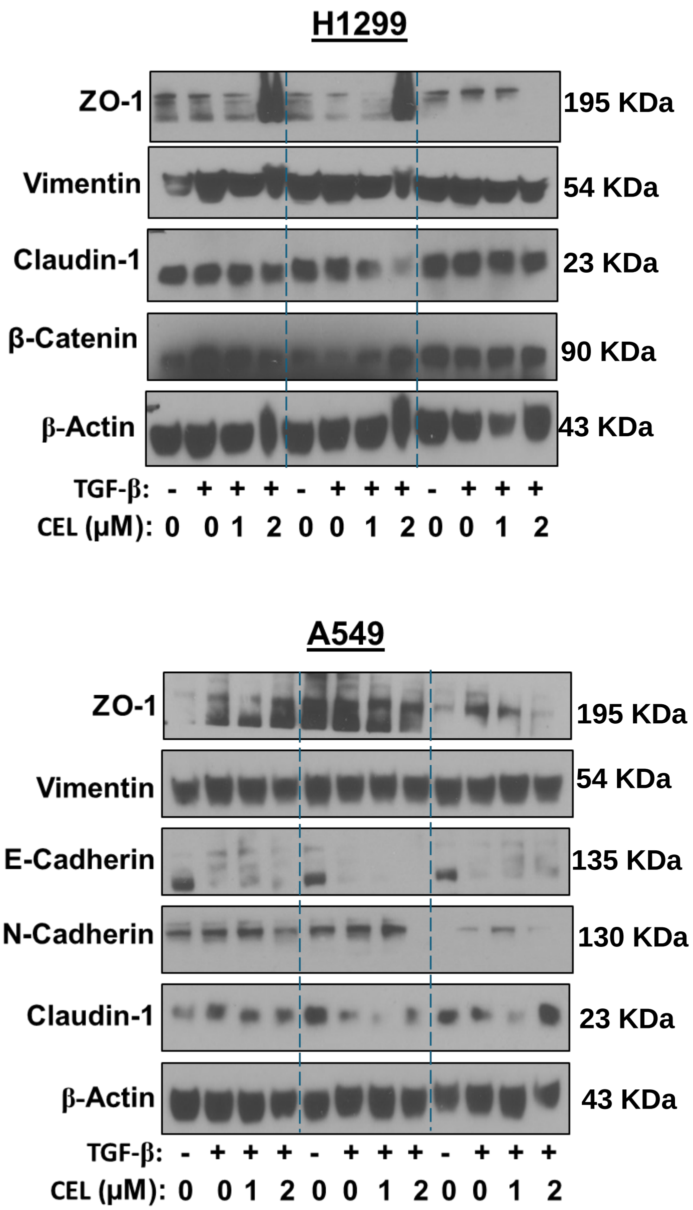

Figure S4. H1299 and A549 cells were treated with TGF-β (5 ng/mL) for 24 h to induce EMT, followed by treatment with CEL (1 or 2 μM) for an additional 24 h. Western blot analysis revealed that CEL reversed TGF-β–induced mesenchymal changes, as shown by upregulation of epithelial markers (ZO-1, E-cadherin, claudin) and downregulation of mesenchymal markers (vimentin, β-catenin, N-cadherin).

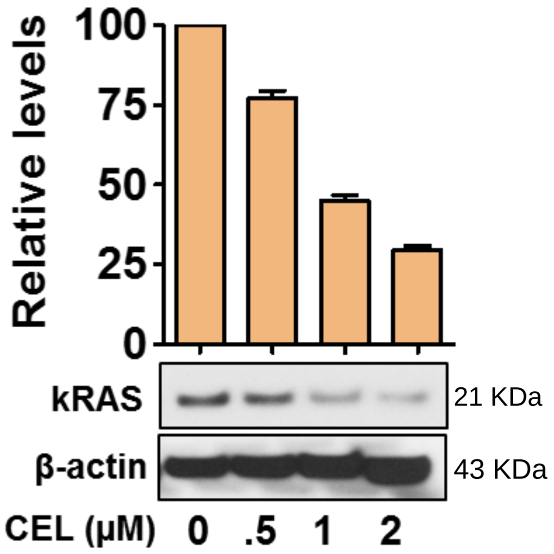

Figure S5. Dose-dependent attenuation of KRAS levels upon CEL treatment in A549 cells.

Table S2. List of primers used in the study.

**Name**	**Sequence**
ZO-1 F	AGAAGATAGCCCTGCAGC
ZO-1 R	AGTCCATAGGGAGATTCC
GAPDH-F	TTGTCAGCAATGCCTCCTGC
GAPDH-R	TCGCTGTTGAAGTCGCAGG
Smad2-F	GCCATCACCACTCAAAACTGT
Smad2-R	GCCTGTTGTATCCCACTGATCTA
TGFB1 F	CAAGCAGAGTACACACAGCAT
TGFB1 R	TGCTCCACTTTTAACTTGAGCC
VIMENTIN F	GCAAAGATTCCACTTTGCGT
VIMENTIN R	GAAATTGCAGGAGGAGATGC
B-ACTIN F	ATCAAGATCATTGCTCCTCCTGAG
B-ACTIN R	CTGCTTGCTGATCCACATCTG
B-CATENIN F	CACAAGCAGAGTGCTGAAGGTG
B-CATENIN R	GATTCCTGAGAGTCCAAAGACAG
CLDN-1 F	GTCTTTGACTCCTTGCTGAATCTG
CLDN-1 R	CACCTCATCGTCTTCCAAGCAC
KRAS F	CAGTAGACACAAAACAGGCTCAG
KRAS R	TGTCGGATCTCCCTCACCAATG
AKT F	TGGACTACCTGCACTCGGAGAA
AKT R	GTGCCGCAAAAGGTCTTCATGG
PI3K F	GAAGCACCTGAATAGGCAAGTCG
PICK R	GAGCATCCATGAAATCTGGTCGC
cMYC F	CCTGGTGCTCCATGAGGAGAC
cMYC R	CAGACTCTGACCTTTTGCCAGG
Smad4 F	CTACCAGCACTGCCAACTTTCC
Smad4 R	CCTGATGCTATCTGCAACAGTCC
E-cadherin F	GCCTCCTGAAAAGAGAGTGGAAG
E-Cadherin R	TGGCAGTGTCTCTCCAAATCCG
N-cadherin F	CCTCCAGAGTTTACTGCCATGAC
N-cadherin R	GTAGGATCTCCGCCACTGATTC
